# Next generation yellow fever vaccine induces an equivalent immune and transcriptomic profile to the current vaccine: observations from a phase I randomised clinical trial

**DOI:** 10.1016/j.ebiom.2024.105332

**Published:** 2024-09-17

**Authors:** Anke Pagnon, Christophe Carre, Marion Aguirre, Emilie Chautard, Sophie Gimenez, Franck Raynal, Emmanuel Feroldi, Paul Scott, Kayvon Modjarrad, Manuel Vangelisti, Nathalie Mantel

**Affiliations:** a Vaccine Research and Development, Sanofi, Marcy l’Etoile, France; b Emerging Infectious Diseases Branch, Walter Reed Army Institute of Research, Silver Spring, MD, USA

**Keywords:** Yellow fever virus, Serum-free, Live-attenuated vaccine, Phase I clinical trial

## Abstract

**Background:**

Yellow fever (YF), a mosquito-borne acute viral haemorrhagic illness, is endemic to many tropical and subtropical areas of Africa and Central and South America. Vaccination remains the most effective prevention strategy; however, as repeated outbreaks have exhausted vaccine stockpiles, there is a need for improved YF vaccines to meet global demand. A live-attenuated YF vaccine candidate (referred to as vYF) cloned from a YF-17D vaccine (YF-VAX®) sub-strain, adapted for growth in Vero cells cultured in serum-free media, is in clinical development. We report the innate and adaptive immune responses and the transcriptome profile of selected genes induced by vYF.

**Methods:**

Healthy adults aged 18–60 years were randomised at a 1:1:1:1 ratio to receive one dose of vYF at 4, 5 or 6 Log CCID_50_ or YF-VAX (reference vaccine), administered subcutaneously in the upper arm (ClinicalTrials.gov identifier: NCT04142086). Blood/serum samples were obtained at scheduled time points through 180 days (D180) post-vaccination. The surrogate endpoints assessed were: serum cytokine/chemokine concentrations, measured by bead-based Multiplex assay; peripheral blood vYF-specific IgG and IgM memory B cell frequencies, measured by FluoroSpot assay; and expression of genes involved in the immune response to YF-17D vaccination by RT-qPCR.

**Findings:**

There was no increase in any of the cytokine/chemokine concentrations assessed through D14 following vaccination with vYF or YF-VAX, except for a slight increase in IP-10 (CXCL10) levels. The gene expression profiles and kinetics following vaccination with vYF and YF-VAX were similar, inclusive of innate (antiviral responses [type-1 interferon, IFN signal transduction; interferon-stimulated genes], activated dendritic cells, viral sensing pattern recognition receptors) and adaptive (cell division in stimulated CD4+ T cells, B cell and antibody) immune signatures, which peaked at D7 and D14, respectively. Increases in vYF-specific IgG and IgM memory B cell frequencies at D28 and D180 were similar across the study groups.

**Interpretation:**

vYF-induced strong innate and adaptive immune responses comparable to those induced by YF-VAX, with similar transcriptomic and kinetic profiles observed.

**Funding:**

10.13039/100004339Sanofi.

**Research in context**

**Evidence before this study**

Live-attenuated yellow fever (YF) vaccines based on the 17D strain have been central to the prevention and management of YF in endemic countries and particularly during outbreaks over the past decades. However, increased demand for YF immunisation has led to global YF vaccine shortages, exacerbated, in part, by insufficient availability of pathogen-free embryonated eggs required for timely vaccine production. We searched PubMed on July 08, 2024, without date or language restrictions, using the search term “Yellow Fever Vaccine” for preclinical or clinical studies of any live-attenuated YF vaccine candidate (referred to as vYF) cloned from a YF-17D vaccine (YF-VAX) sub-strain adapted for growth in Vero cells cultured in serum-free media. Three preclinical studies were identified. The available data suggests that the vYF candidate may be less neurovirulent than the marketed YF-17D vaccines, Stamaril and YF-VAX, but would have similar viscerotropism, immunogenicity and efficacy.

**Added value of this study**

This first-in-human study shows that vYF, across all doses assessed, induces strong innate antiviral responses and adaptive B cell responses similar to those seen with YF-VAX. The targeted transcriptomic profiles and kinetics after vaccination with vYF involved the same genes and pathways as those elicited by vaccination with YF-VAX, demonstrating similar responses to both vaccines. These similarities may suggest that the immunity conferred by vYF may be of similar efficacy and longevity to that of YF-VAX. The vYF vaccine, therefore, is a promising candidate to advance toward large-scale clinical testing.

**Implications of all the available evidence**

The available evidence suggests that vYF provides similar immunity to that with YF-VAX. Future clinical trials should aim to confirm the safety and immunogenicity of vYF in large numbers of participants from different regions, including children, to support licensure of the candidate vaccine.

## Introduction

Yellow fever (YF), a mosquito-borne acute viral haemorrhagic illness caused by the yellow fever virus (YFV), remains a major global health concern. YF has three transmission cycles (jungle, savannah, and urban) and is endemic to many tropical and subtropical areas of Africa and South America; however, sub-Saharan Africa bears 90% of the burden.^1^, ^2^, ^3^ Signs, symptoms and outcomes of severe illness include fever with epigastric pain, hepatitis with jaundice, renal failure, haemorrhagic sequelae and shock,^1^^,^^4^ resulting in death in 20–60% of confirmed cases.^1^^,^^5^ Recent figures from 2018 estimate that YFV causes 109,000 severe infections and 51,000 deaths annually in Africa and South America.^6^

YF is a vaccine-preventable disease, with licensed vaccines providing long-lasting protective immunity after a single dose in the majority of vaccine recipients,^7^ though waning of immunity has been reported in young infants.^8^ Neutralising antibody titres following YF vaccination are generally considered (and recommended by the World Health Organization TRS 978 annex 5)^9^ as a correlate of protection,^10^ as they have been experimentally validated as such in non-human primate (NHP) models.^11^ YF-17D vaccines induce rapid, antigen-specific IgM neutralising antibody responses that can be detected from about 7 days post-vaccination, peaking at 2–3 weeks before subsiding to baseline over the course of several months, whereas IgG neutralising antibodies develop more slowly, peaking 4–6 weeks post-vaccination and persisting for up to 40 years.^10^^,^^12^^,^^13^ Cellular immune mechanisms, including CD4^+^ and CD8^+^ T cell responses, also contribute to long-lasting protective immunity^13^; indeed, *in vitro* expandable T cells could be detected more than >10 years post-vaccination in >90% of YF-17D recipients.^14^ Early innate immune response to YF-17D vaccine also may play a role in determining the strength/magnitude and functional quality (breadth, avidity, polyfunctionality, memory phenotype, or durability) of the adaptive immune response as dendritic cells play a vital role in initialising and optimising the adaptive immune response by sensing microbial stimuli through pattern-recognition receptors.^12^^,^^15^^,^^16^

A live-attenuated YF vaccine candidate (referred to as vYF) cloned from a YF-17D vaccine (YF-VAX) sub-strain, adapted for growth in Vero cells that have been cultured in serum-free media, is currently in development. vYF has previously been shown to induce robust neutralising antibody responses in hamsters (range 3.2–5.2 Log μPRNT_50_) that far exceeded the threshold considered to be protective (i.e. 10 PRNT_50_).^17^ In addition, vYF induced robust immunogenicity in cynomolgus macaques, at least at a magnitude similar to that achieved with the two licensed YF-17D vaccines (YF-VAX and Stamaril) and protected against challenge with wild-type YFV, Asibi strain.^18^

The first-in-human phase I clinical trial of this same vYF candidate—a randomised, observer-blind, dose-ranging (4, 5, and 6 Log CCID_50_), YF-VAX-controlled study in US adults (n = 72) showed that vaccination yielded favourable safety and immunogenicity profiles comparable to those observed with YF-VAX.^19^ There were no serious adverse events in any study group and no associated safety concerns. In general, solicited injection site and systemic reactions were less frequently reported with vYF compared to YF-VAX. The candidate vaccine induced robust neutralising antibody responses that peaked at 28 days post-vaccination and persisted well above the established protective threshold through 6 months of follow-up. In the current report, we evaluated the innate immune response, the transcriptome profile of selected genes, and the specific memory B cell response induced by vYF in participants from the phase I clinical trial as surrogate markers of efficacy.

## Methods

### Study design

The design for this randomised, observer-blind, dose-ranging, YF-VAX-controlled phase I clinical trial (ClinicalTrials.gov Identifier: NCT04142086) has been described in a separate report.^19^ Healthy adults aged 18–60 years were eligible for inclusion. Eligibility was not contingent on YF serostatus; however, participants with a known history of prior vaccination for a flavivirus disease or prior known flavivirus infection were not eligible for participation. Other exclusion criteria included pregnancy, lactation or not using an effective method of contraception for at least 4 weeks prior to and after vaccination, if of childbearing potential; participation or planned participation in another clinical trial at the time of study enrolment (or in the 4 weeks preceding the trial vaccination); receipt or planned receipt of any vaccine in the 4 weeks preceding or following the trial vaccination; receipt of immune globulins, blood or blood-derived products in the prior 6 months; personal or family history of thymic pathology (thymoma, thymectomy, or myasthenia); known history of hepatitis B or hepatitis C seropositivity; current alcohol abuse or drug addiction; known or suspected human immunodeficiency virus infection; known or suspected congenital or acquired immunodeficiency; receipt of immunosuppressive therapy, such as anti-cancer chemotherapy, or radiation therapy, within the preceding 6 months; or long-term systemic corticosteroid therapy (prednisone or equivalent for more than 2 consecutive weeks within the past 3 months); known systemic hypersensitivity to any of the vaccine components, eggs, or history of a life-threatening reaction to the vaccines used in the trial or to a vaccine containing any of the same substances; chronic illness; moderate or severe acute illness or infection; receipt of any anti-viral in the 2 months preceding vaccination and up to 6 weeks after; and planned travel to YF endemic countries within 6 months of vaccination; deprived of freedom by an administrative or court order, or in an emergency setting, or hospitalised involuntarily; immediate family members of the Investigators or employee with direct involvement in the proposed study. Participants were enrolled at Walter Reed Army Institute of Research (WRAIR) Clinical Trials Center (Silver Spring, Maryland, USA) between January 15, 2020 and June 24, 2021 and randomised using interactive response technology in a 1:1:1:1 ratio to receive one subcutaneous dose of vYF at 4, 5, or 6 Log CCID_50_ or YF-VAX in the upper arm. The study vaccinator oversaw preparation and administration of the product and was not authorised to collect any safety data. The investigators, sponsor, laboratory staff and all participants were blind to vaccine allocations before database lock. vYF was grown in a proprietary serum-free Vero cell line, derived from a commercially available Vero cell line (cat# CCL-81; ATCC, Manassas, VA, USA). Blood/serum samples were obtained at multiple time points pre- and post-vaccination.

### Study outcomes

The co-primary outcomes of this phase I study were safety, immunogenicity and YF viraemia following vaccination with one of three vYF doses or YF-VAX have been previously published.^19^ The observational outcomes, reported here, involved the assessment of the inflammatory and innate immune response biomarkers and the cellular immune response following vaccination with one of three vYF doses or YF-VAX.

### Luminex for cytokine quantification

All participants provided a pre-vaccination serum sample at baseline (Day 0 [D0]) and post-vaccination serum samples on D1, D3, D5, D7, D10, and D14 for pro-inflammatory cytokine quantification. Serum cytokine concentrations were analysed using two human Milliplex MAP Kits (Merck Millipore, Darmstadt, Germany): the human cytokine/chemokine/growth factor panel A magnetic bead panel for 15 cytokines (IFNγ, TNFα, IL1α, IL1β, IL5, IL6, IL8, IL10, IL12p40, IL17α, IL18, G-CSF, MIP1α, MCP1, IP10 [CXCL10]), and CCL5/RANTES, according to the manufacturer's instructions. The serum samples were added (undiluted for 15-plex and 100-fold diluted for RANTES) to a mixture of colour-coded-magnetic beads, pre-coated with analyte-specific capture antibodies. Streptavidin-phycoerythrin conjugates were added to analyte-specific biotinylated antibodies, and the fluorescent signals were measured using the multiplex array reader Bio-Plex 200 System (Bio-Rad Laboratory, Inc, Hercules, CA, USA). The median fluorescence intensities (MFI) were automatically analysed using a 5-parameter logistic regression standard curve for calculating analyte concentrations in samples (pg/mL). The ratios versus the pre-vaccination time point (D1/D0, D3/D0, D5/D0, D7/D0, D10/D0, D14/D0) were also calculated to evaluate the fold-rise over baseline of the post-vaccination pro-inflammatory response.

### Memory B cell IgG and IgM FluoroSpot assay

All participants provided a pre-vaccination blood sample at baseline (D0) and post-vaccination on D28 and on D180 for memory B cell assessments. Memory B cells secreting vYF-specific IgG and IgM antibodies were quantified using a FluoroSpot Assay (FluoroSpot; Mabtech AB, Stockholm, Sweden) according to manufacturer's instructions.

Peripheral blood mononuclear cells (PBMCs) were isolated and cryopreserved until analysis. Cryopreserved PBMCs were thawed and cultured (only samples with viability >70%) for 5 days in RPMI supplemented with Human memory B-cell StimPack (Mabtech AB, Stockholm, Sweden) to induce terminal differentiation into antibody secreting cells (ASCs).

FluoroSpot plates (Mabtech AB, Stockholm, Sweden) were coated with anti-vYF monoclonal antibody HB112-4G2 (in-house mAb; cat# HB-112™; ATCC, Manassas, VA, USA) for vYF-specific responses, or mouse anti-IgG/IgM mAbs (Mabtech AB, Stockholm, Sweden) for total IgG/IgM response; the specificity and reactivity of these antibodies has been previously validated.^20^^,^^21^ After overnight incubation at 5 °C, washing and blocking, the vYF vaccine was then added to the vYF-specific wells (10.76 Log GEq/mL, Genomic Equivalents per mL), except for negative controls (HB112-4G2 mAb only) and incubated for 4 days at 5 °C. Then, serial dilutions of stimulated PBMCs were added to vYF-specific wells, or to total IgG wells, for 5 h and removed. The following day, plates were incubated for 2 h at 20 °C with respective fluorochrome-labelled detection antibody anti-IgG 550 (clone MT78/145; Human IgG FluoroSpot FLEX kit, cat# FSX-05R-10, Mabtech AB, Stockholm, Sweden) or anti-IgM 640 (clone MT22; Human IgM FluoroSpot FLEX kit, cat# FSX-17M, Mabtech AB, Stockholm, Sweden). The plates were finally washed and dried at ambient temperature (protected from light), before counting IgG and IgM spots (ASCs) with the FluoroSpot reader (ISpotSpectrum, AID, Strassberg, Germany).

For each sample and each isotype, vYF-specific ASC/10^6^ cells (after subtraction of 4G2 mAb ASC/10^6^ cells) and total ASC/10^6^ cells were calculated to assess the frequency of IgG and IgM vYF-specific responses. The vYF-specific responses were calculated by dividing the respective number of vYF-specific IgG or IgM ASC/10^6^ cells by the total IgG or IgM ASC/10^6^ cells. The D28/D0 and D180/D0 ratios were calculated to evaluate the fold-rise over baseline of the specific and total responses at 28- and 180-days post-vaccination, respectively.

### Whole blood RNA transcriptomic analysis of selected genes by RT-qPCR

The expression of 85 genes (Supplementary Table S1) previously shown to be involved in the immune response to YF-17D vaccination in NHPs^18^ (and humans^22^, ^23^, ^24^, ^25^), as well as 5 housekeeping genes (*NONO*, *GUSB*, *SDHA*, *HPRT1*, and *UBE2D2*), were assessed by RT-qPCR assay on the Fluidigm digital array platform using a customised set of 90 TaqMan Gene Expression Assays (Thermo Fisher Scientific, Waltham, MA, USA) for the corresponding selected genes (Supplementary Table S1). The criteria used to select this panel of genes for immuno-profiling by RT-qPCR were: 1) genes clearly involved in the immune response based on the literature (all genes with unknown or unclear function were not selected); 2) NHP genes without known orthologues in humans were not selected; 3) genes up or down regulated in both humans and NHPs or in human only (to avoid elimination of human differentially expressed gene [DEG] since this was our final target, earlier kinetics are observed in NHPs; we may have missed the timepoint where adaptative response initiation was observed [D14 in human]); 4) all the genes differentially expressed only at D21 and/or D28 post-vaccination were not selected.^25^ For the later criteria, at D21 and/or D28 post-vaccination timepoints, the activated pathways were not considered linked to vaccination (these likely reflect renewal of erythrocytes following frequent blood sampling). In addition, the genes that encoded the heavy constant chain of immunoglobulins IgG1, IgG2, IgG3, IgG4, and IgM were added to the list of genes selected to assess their expression level as these were previously shown to be involved in the immune response to YF vaccination and could be a marker of B cells and humoral response.^24^ Although numerous potential immunoglobulin genes were identified in the BioVacSafe human study based on the annotations of the probes identified as DEGs at D14,^25^ the selection was focused on heavy constant chains to maximise the chance of detecting the expression of immunoglobulin while reducing the number of targets to design. *TLR3* was also identified in all NHPs but not in humans. However, since it was identified as a key component of the immune response to the vaccine in NHPs, we decided to add it to the panel.

In brief, whole blood was collected in PAXgene tubes (PreAnalytix, Qiagen-BD Company, Hombrechtikon, Switzerland) at baseline and at D1, D3, D5, D7, D10, D14, D28, and D180, and stored according to the manufacturer's protocol prior to RNA extraction. Total RNA was extracted with the PAXgene 96 Blood RNA kit (PreAnalytix, Qiagen-BD Company, Hombrechtikon, Switzerland) according to manufacturer's instructions on the Tecan Evo 150 workstation (Tecan, Männedorf, Switzerland). RNA samples were quantified by spectrophotometry and stored at −80 °C until further processing and assessment. Potential residual genomic DNA in RNA samples was eliminated by a Turbo DNase step followed by an RNA clean-up and concentration step on silica column (Clean and concentrator; Zymo Research, Irvine, CA, USA). The RNA quantity, purity, and quality were checked on the TapeStation (Agilent Technologies, Santa Clara, CA, USA) and the DropSense 96 or Lunatic (Unchained Labs, Pleasanton, CA, USA). Samples with A260/A280 ratio ≈2, RNA Integrity Number (RIN) ≥6, and minimal RNA concentration of 50 ng/μL were used for the next steps. Residual genomic DNA contamination was quantified by qPCR Femto™ assay (Zymo Research, Irvine, CA, USA) and had to be <5pg/μL after the final dilution.

The RNA extracted from the samples was reverse-transcribed using the Reverse Transcription Master Mix kit (Standard Biotools, San Francisco, CA, USA) according to the manufacturer's instructions. Each cDNA sample was preamplified using TaqMan PreAmp Master Mix 2X kit (Applied Biosystems, Bedford, MA, USA) and a pool of the 90 TaqMan pairs of primers to increase the number of copies of target DNA. The qPCR step was performed in a Fluidigm BioMark qPCR thermocycler (Standard Biotools, San Francisco, CA, USA) with a TaqMan Universal PCR Master Mix kit (Applied Biosystems, Bedford, MA, USA). For each sample, two independent RT-qPCR runs were performed. At each step, we included positive and negative controls. Positive controls were prepared using blood from a flavivirus-naïve subject (Blood donation from Etablissement Français du Sang [EFS], France), and negative control consisted of nuclease-free water. We also included: extraction positive (TextP) and negative (TextN) controls, RT step positive (TrtP) and negative (TrtN) controls, preamplification positive (TpAmpP) and negative (TpAmpN) control, PCR non-template control (NTC), PCR non-reagent control (NRC, without the detection probe), and no RT control (TnoRT).

C_t_ values were computed using Fluidigm Real-Time PCR analysis software (Standard Biotools, San Francisco, CA, USA) and normalised using the fluorescence of ROX passive reporter (Raw Taqman—Background Taqman)/(Raw ROX—Background ROX). Fluidigm Biomark™ quality thresholds, based on amplification curves, were used at the default 0.65 threshold value. Overall, only 0.2% of C_t_ values located outside the C_t_ optimal 6–25 range recommended by Fluidigm were considered undetectable and coded at 25 (no values observed <6). Almost all NRCs and other negative control samples (NTC, TpAmpN, TrtN, TextN, TnoRT) were undetectable, and almost all positive controls (TpAmpP, TrtP, TextP) were detected, except for 11 target genes (*CCL2*, *IFI6*, *IGHG3*, *IRF7*, *ISG15*, *KCTD14*, *LY6E*, *PARP9*, *SDHA*, *TRIM22*, *XAF1*) having more than 25% flagged reactions in at least one plate that were excluded from all analyses. Missing values for 33 flagged reactions were imputed using the target mean.

Thirty-five out of 595 samples were not analysed because they were either lost (broken tubes [n = 6]) or had poor quality mRNA (RNA concentrations <50 ng/μL, TapeStation RINe <6 [n = 29]). For samples having a genomic DNA (gDNA) quantity over the threshold based on Femto qPCR assay (i.e. >5 pg/μL after the final dilution step), an additional control was added by testing the RNA directly without the reverse transcriptase (RT) step (no RT control) to ensure that the residual gDNA was not detected with the different qPCR reactions. Samples from one participant with a very high proinflammatory profile at baseline were excluded as outliers. This participant also showed high IP10 [CXCL10] concentration in serum at baseline. No sample (other than that mentioned) appeared as a potential outlier, before and after normalisation, when checked by scaled principal component analysis (PCA) and hierarchical clustering using all the target genes. The quality control was completed by density and correlation plots of all samples.

A two-step delta–delta C_t_ (DDCt) normalisation was applied to each assay plate, using 4 of the 5 predefined housekeeping genes (these genes showed only limited variation at only one time point) and 3 human sample TrtP controls (these control samples were not different from those of study participants at baseline and clustered with them). The assay plates were replicated in pairs with high reliability (intraclass correlation coefficient >0.9 in a prior technical pilot study), batch corrected using the Combat parametric algorithm (sva bioconductor package, version 3.42.0)^26^ and summarised by the mean of the pair of values, except for one sample (participant #19 baseline sample) where the first run (but not the second) gave very different results from those of the other participants, suggesting a potential issue. As the gene expression for each time point for each participant was normalised compared to their baseline, to avoid introduction of a “run effect” bias, all time points for the same participant were assessed on the same plate (i.e. treated at the same time, with the same operator and same reagent batches).

### Ethics

The study was conducted in compliance with the International Conference on Harmonisation guidelines for Good Clinical Practice and the principles of the Declaration of Helsinki, as well as local and/or national regulations and directives. The protocol and amendments (WRAIR 2689, HRPO Log E00378.6a-EID001) were approved by the WRAIR Institutional Review Board and applicable local and/or national regulatory agencies. Written informed consent was obtained from participants before study enrolment.

### Statistical analysis

All analyses for this publication were performed on the full analysis set (FAS). The FAS consisted of all participants who received one dose of vYF or YF-VAX and had at least one valid post-vaccination blood sample result.

vYF-specific ASC frequencies were evaluated by descriptive statistics at the timepoints assessed: geometric means (GM) of vYF-specific IgG and IgM ASC frequencies, respective geometric mean ratios (GMR) at each post-vaccination timepoint versus pre-vaccination (D0) and associated 95% CIs.

Time course inferential statistical analyses for differences between vaccines and vaccine doses were implemented using the empirical Bayesian approach of Ritchie et al. 2015 (limma Bioconductor package, version 3.50.3),^27^ which provides more robust estimates of DEGs by borrowing the gene variance estimation across all the gene targets. Repeated time point measurements for participants were handled according to the usual method of the limma package.

Normality of the DDCt values was tested by group and gene using Shapiro Wilk test leading to a majority of non-significant p values (additionally the whole nonparametric analyses were performed showing similar test conclusions compared to their parametric counterparts). Two series of longitudinal analyses were presented to handle possible effects of baseline imbalance between vaccines and dose groups: absolute time points and change versus baseline (D0). Group differences, for both absolute change and change against baseline series, were tested by adding the interaction term “group with time point” into the model, and by further testing adequate contrasts: e.g. (DDCt D7—DDCt D0)_YF-VAX_—(DDCt D7–DDCt D0)_vYF_all_doses_ for the change from baseline (second series). To correct for multiple testing in the linear model comparisons, Benjamini and Hochberg's False Discovery Rate (FDR) procedure was applied. The inferential analysis results were presented using heatmaps with hierarchical clustering, and additionally with individual participant time course curves.

PCA was computed using prcomp R function, and scaled as gene expression exhibits various ranges, and hierarchical clustering was computed using complete linkage method with Euclidean distance. UpSet plot was produced using R package ggupset version 0.2.1.

### Role of funders

Sanofi funded this study and was involved in the study design, sample analysis, and interpretation of data, the writing of the report; and in the decision to submit the paper for publication. All authors had full access to all the data in the study and had final responsibility for the decision to submit for publication.

## Results

### Study participants

A total of 73 participants were enrolled and randomised; however, one participant did not receive a study vaccine on D0 due a technical issue. As such, 72 participants received one subcutaneous dose of 4, 5, or 6 Log CCID_50_ vYF or YF-VAX (18 per group) and had at least one valid post-vaccination blood sample result. The characteristics of the participants in the FAS have been presented in a separate manuscript.^19^ Overall, the majority of participants were male (56.2%; 41/73) and Caucasian/white (53.4%; 39/73), with a mean age (standard deviation) of 35.4 (11.2) years. Across the study groups, 11–22% were YF seropositive at baseline, as determined by YF microneutralisation assay (titre ≥10).

### Systemic cytokines/chemokines

**Fig. 1:**
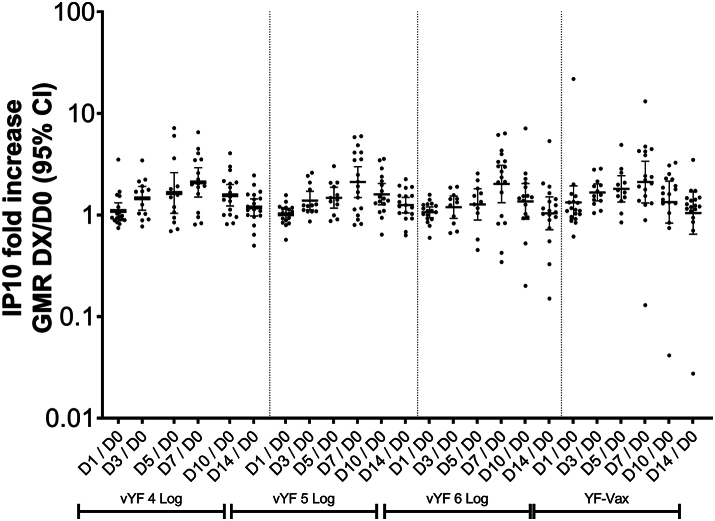
Serum IP-10 (CXCL10) concentration following vaccination with vYF (4, 5 and 6 Log CCID_50_) and YF-VAX (FAS; n = 72). Data shown for individual participants, with the geometric mean indicated by the black line, and lower and upper black lines representing the 95% confidence interval.

After vaccination with vYF (4, 5, and 6 Log CCID_50_) or YF-VAX there was no increase in the serum concentration of the 16 cytokines/chemokines assessed through to D14 (Supplementary Table S2), except for IP-10 (CXCL10), where an approximate 2-fold increase was observed from D1 to D7 followed by a decrease to baseline by D14 (Fig. 1). The changes in IP-10 levels were not significantly different between the vYF groups or compared with the YF-VAX group.

### Targeted transcriptomic responses

**Fig. 2:**
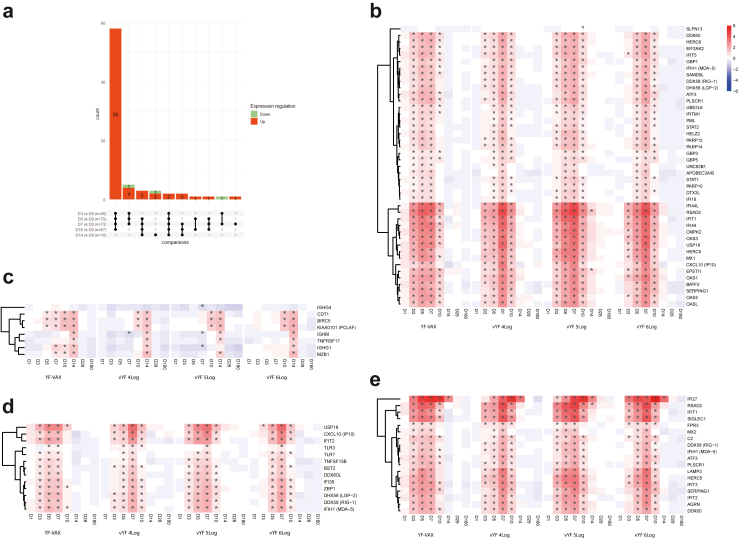
UpSet plots of differentially expressed genes for each time point versus the baseline, and the number of genes in common for each comparison across the vaccine groups (FAS; n = 72) (a). Genes are considered in common if they have the same fold change direction. In the bar plot, red represents up-regulated and green down-regulated genes. Heatmaps of selected differentially expressed genes after vaccination at each time point versus baseline, grouped by functional category: (b) antiviral innate response (type I interferon signature, IFN signal transduction/ISG); (c) adaptive immune response (cell division of stimulated CD4+ T cells, B cells and antibody response); (d) viral sensing; and (e) activated dendritic cells.

The expression profiles of the genes involved in early immune responses to vaccination with vYF (irrespective of dose) and YF-VAX were similar (Fig. 2). The innate immune response signatures (antiviral responses [type 1 IFN, IFN signal transduction; IFN-stimulated genes], activated dendritic cells, and viral sensing pattern recognition receptors) peaked at D7 across the vYF and YF-VAX groups. Those for the adaptive immune response (cell division in stimulated CD4+ T cells [*CDT1*, *BIRC5*, and *KIAA0101* (PCLAF)], B cell and antibody response [*IGHM*, *IGHG1*, *MZB1*, and *TNFSF17*]) peaked at D14. One participant from the YF-VAX group was excluded from the statistical analysis on a biological basis, as they exhibited a very high inflammatory response at baseline. Nevertheless, this participant's immune response after vaccination followed a similar expression profile as the others, with activation of the same pathways.

The expression kinetics of the selected DEGs relative to baseline are presented as heatmaps in Fig. 2b for between vaccine differences and between vYF dose differences, respectively. The significant gene expression changes from baseline for all groups appeared similar (same up- or down-regulated DEG profiles), with no significant (absolute and versus baseline) differences between groups in nearly all comparisons. Supplementary Figure S1 shows the individual participant time course for *IFI27* gene expression by vaccine and dose group for illustration purpose. The time course of all genes expressed was assessed, and the *IFI27* gene is presented as an example, as it presented the highest fold change. The UpSet plots in Fig. 2a summarise the overlap of gene expression changes shared by the vaccine groups across time points. It shows that a majority of DEGs were up-regulated and their response was maintained for all timepoints between D3 and D10. Although no significant differences appeared between groups, a slight kinetic difference was observed in the peak responses (D5 or D7) (Fig. 2b and Supplementary Figure S1).

### IgG and IgM memory B cells

**Fig. 3:**
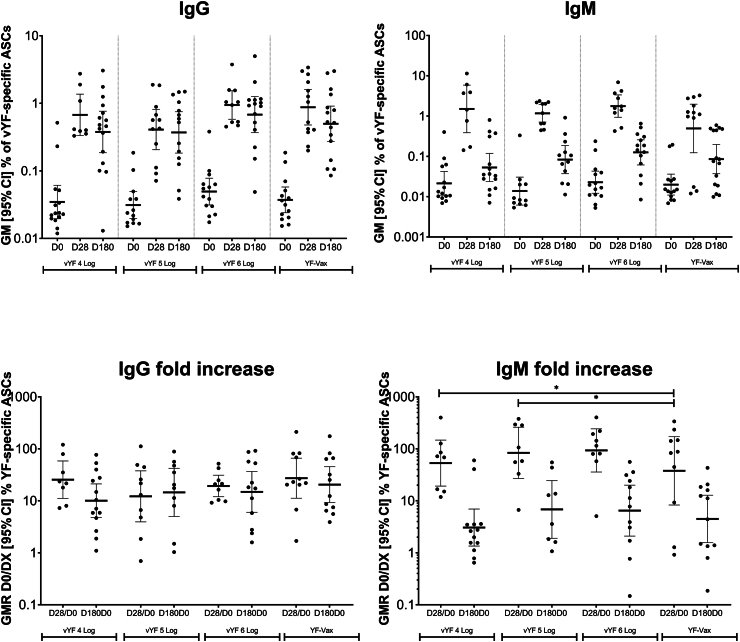
IgG and IgM antibody secreting cell frequencies following vaccination with vYF (4, 5, and 6 Log CCID_50_) or YF-VAX (FAS; n = 72). Data shown for individual participants, with the geometric mean indicated by the black line, and lower and upper black lines representing the 95% confidence interval.

Memory B cell responses were assessed by measuring the proportion (%) of vYF-specific IgG and IgM ASCs versus total IgG and IgM ASC in PBMCs. Overall, there were similar changes in the frequency of IgG and IgM vYF-specific ASCs at D28 and D180 following vaccination with vYF (irrespective of dose assessed) and YF-VAX (Fig. 3).

vYF-specific IgG ASCs were not detected before vaccination (i.e. below the lower limit of quantification) in most (91.1%; 51/56) participants across the study groups. The few exceptions that were vYF-specific IgG ASC positive (>15 ASC/10^6^ PBMCs; n = 5) at baseline likely had previous exposure to one or more flaviviruses (dengue, Zika, YF). After vaccination, there was an increase in the frequency of IgG ASCs at D28, across the study groups, ranging from 0.41% (95% CI, 0.21–0.81) in the 5 Log CCID_50_ vYF group to 0.94% (95% CI, 0.58–1.53) in the 6 Log CCID_50_ vYF group (Fig. 3), with geometric mean D28/D0 ratios ranging from 12 to 27. At D180, the frequency of IgG ASCs remained at levels comparable to those at D28, ranging from 0.37% (95% CI, 0.18–0.75) in the 5 Log CCID_50_ vYF group to 0.68% (95% CI, 0.37–1.26) in the 6 Log CCID_50_ vYF group, with the corresponding geometric mean D180/D0 ratios ranging from 10 to 20. There was no significant difference in the IgG ASC fold increase (D28/D0 and D180/D0) between vYF (all three doses) and YF-VAX (p value ranges from 0.447 to 0.877; linear mixed effect model).

There was also an increase in the frequency of IgM ASCs at D28 across the study groups, ranging from 0.49% (95% CI, 0.12–1.98) in the YF-VAX group to 1.77% (95% CI, 0.94–3.34) in the 6 Log CCID_50_ vYF group, with the corresponding geometric mean D28/D0 ratios ranging from 38 to 93 (Fig. 3). At D180, the frequency of IgM ASCs had decreased to near baseline levels in all study groups, ranging from 0.05% (95% CI, 0.02–0.12) in the 4 Log CCID_50_ vYF group to 0.13% (95% CI, 0.06–0.26) in the 6 Log CCID_50_ vYF group, with the corresponding geometric mean D180/D0 ratios ranging from 3 to 7. The IgM ASC fold increases at D28/D0 for the vYF 4 Log CCID_50_ (p = 0.018; linear mixed effect model) and vYF 5 Log CCID_50_ (p = 0.032; linear mixed effect model) groups were significantly higher than for YF-VAX. No significant differences in IgM ASC fold increases were observed at other time points between vYF and YF-VAX.

## Discussion

We compared the inflammatory response induced by different doses of a vYF vaccine candidate to that with the licensed YF-VAX vaccine, shown to be highly immunogenic and to confer long-term protective immunity after a single dose immunisation. We showed that the immune response induced by vYF vaccination was very similar to that observed with YF-VAX, as measured by three endpoints: i) quantification of 16 pro-inflammatory markers in sera from D0 to D14 (7 time-points); ii) expression profile of 85 genes known to be involved in YF-17D vaccine immunity by RT-qPCR (D0–D14); and iii) quantification of the memory B cell response pre- (D0) and post-vaccination (D28 and D180). Although the mechanism for the durability of YF-VAX is not well understood, these results (based on the similarity of the immunologic and genetic signature profiles and kinetics) suggest that the protective immunity provided by vYF may be of similar longevity to that with YF-VAX.

There was no increase in the cytokines/chemokines assessed, except for IP-10 where a relatively small 2-fold rise was observed at D7 post-vaccination across all vaccine groups. In addition, we observed that IP-10 (CXCL10) was differentially expressed (up-regulated) following vaccination across all vaccine groups. IP-10 is crucial for leukocyte trafficking and homing to inflamed tissue, and has also been found to activate T cells (Th1), NK cells, macrophages/monocytes, dendritic cells and B cells.^28^ Although IP-10 was initially identified as an IFN-γ-inducible protein, no IFN-γ could be detected at the 7 time-points assessed through to D14 post-vaccination in our study. It is possible that the small increase in IP-10 levels observed may have been induced in an IFN-γ-independent manner.^29^^,^^30^

The expression profiles of the genes involved in early immune responses to vaccination with vYF and YF-VAX were similar, with strong antiviral innate immune response signatures that peaked at D7 post-vaccination, and adaptive immune response signatures that peaked at D14. The regulation of gene expression observed in the current study is similar to previous observations in NHPs vaccinated with vYF, Stamaril or YF-VAX^18^^,^^25^ and in published studies of humans vaccinated with YF-17D.^19^^,^^23^^,^^31^ Overall, we observed an antiviral innate immune response signature with strong IFN and viral sensing responses and innate immune cell activation. There were also important overlaps between the up-regulated gene expression profile in our current study and those published by Querec et al.^22^ and Gaucher et al.^23^ for human transcriptional responses following vaccination with YF-17D. For example, several genes associated with innate immunity and interferon responses, such as *IFIT2*, *RSAD2*, *OAS3*, *OASL*, *DDX58* (RIG-1), and *IFIH1* (MDA-5), as well as the key transcription factors *EIF2AK2* and the E3 ubiquitin ligase *HERC5* and *HERC6*, were upregulated at D7 after vaccination in both our trial and the Querec et al. study.^22^

The onset of gene transcription activation occurred at D5 and D7 post-vaccination in the 5 Log CCID_50_ vYF and YF-VAX groups, and slightly later (∼D7) in the 4 and 6 Log CCID_50_ vYF groups. This phenomenon cannot be explained by virus dose injected, nor by egg-matrix effects as the 5 Log CCID_50_ vYF dose group behaved similarly to the YF-VAX group. Nevertheless, the transcriptomic profile after vaccination with vYF at all three doses were the same genes and pathways as vaccination with YF-VAX, demonstrating similar responses to both vaccines.

In general, the kinetics of the IgG and IgM ASC frequencies across the three vYF doses and YF-VAX were generally similar. Both IgG and IgM ASCs were induced at D28 post-vaccination, with higher IgM than IgG ASC frequency. IgG ASC frequency, however, persisted more through D180, while IgM ASC frequency decreased to near baseline level. There was no significant difference in the IgG and IgM ASC-fold increase (D28/D0 and D180/D0) between vYF (all three doses) and YF-VAX, except for significantly higher IgM ASC-fold increases at D28/D0 in the 4 and 5 Log CCID_50_ vYF groups than the YF-VAX group. These ASC kinetic profiles are in concordance with a recent study in humans that showed that classic IgM + CD27+ B cells waned rapidly,^32^ whereas switched immunoglobulin (swIg+) and atypical IgM+ and IgD + memory B cell were stable over time. We also showed in a NHP model,^18^ sustained memory B cell frequencies in peripheral blood, from D14 through to D221 after vYF vaccination, similar to that observed after vaccination with Stamaril and YF-VAX. In that animal study, we identified the same trend for both IgM and IgG memory B cells, in contrast to the current study, where the IgM memory B cell response returned to baseline at D180. The NHP study also showed that both IgM and IgG memory B cells were induced at D14 after vaccination and expanded up to D90 post-vaccination. At D221 post-vaccination, both IgM and IgG memory B cell frequencies remained high: about 1% of IgM and 0.1% of IgG memory B cells were still detectable in peripheral blood.

This study had a few limitations that warrant discussion. Firstly, the study had a small sample size; however, this is not uncommon for first-in-human phase I studies. Secondly, access to more post-vaccination immune cell samples would allow for deeper analysis of the immune cell populations, including T-cells, by advanced flow cytometry analysis, further adding to existing knowledge on the immune response to YF vaccination. Robust, long-lived and polyfunctional T cell immune responses are also elicited following vaccination with YF-17D vaccines, though their role in protection against subsequent challenge remains unclear.^33^ Lastly, race and ethnicity profiles of participants was consistent with that of the US army but may not necessarily be representative of the overall US population or the population likely to use the vaccine worldwide.

In conclusion, vYF induces strong innate antiviral responses and adaptive B cell responses similar to those elicited by YF-VAX, with no major differences between the vYF doses assessed. The transcriptomic profile of selected genes and kinetics after vaccination with vYF at all three doses were the same genes and pathways as vaccination with YF-VAX, demonstrating similar responses to both vaccines, which may suggest that the immunity provided with vYF would be of similar longevity to that with YF-VAX. The immunogenicity and safety profile of vYF compared with licensed yellow fever vaccines will be assessed in subsequent phase II/III studies.

## Contributors

AP, CC, MA, EC, FR, EF, PS, MK, MV, and MN contributed to the concept or design of the study; AP, CC, MA, SG, FR, and MN were involved in data acquisition; and AP, CC, MA, EC, SG, FR, MV, and MN were involved in the analysis or interpretation of the data. AP, CC, MA, EC, SG, FR, MK, and MN were involved in drafting the initial manuscript. All authors critically revised the manuscript, approved the final version for publication and are accountable for its accuracy and integrity. All authors accessed and verified the underlying data.

## Data sharing statement

The datasets generated and/or analysed during the current study, including the raw data, are not publicly available in order to safeguard the privacy of participants and the confidentiality and protection of their data, as well as protect commercially sensitive information. Qualified researchers may request access to participant level data and related study documents including the clinical study report, study protocol with any amendments, blank case report form, statistical analysis plan, and dataset specifications. Participant level data will be anonymised and study documents will be redacted to protect the privacy of our trial participants. Further details on Sanofi's data sharing criteria, including required permissions to access the data, eligible studies, and process for requesting access can be found at: https://www.vivli.org/.

## Declaration of interests

AP, CC, MA, EC, SG, FR, EF, MV, and NM are Sanofi employees and hold shares and/or stock options in the company. PS and KM received funds from Sanofi through their institution (WRAIR) to support their work in the vYF01 trial.

The opinions expressed herein are those of the authors and should not be construed as official or representing the views of the US Department of Defense or the Department of the Army.
