# Supplementary material for: Next generation yellow fever vaccine induces an equivalent immune and transcriptomic profile to the current vaccine: observations from a phase I randomised clinical trial
Source: eBioMedicine. 2024 Sep 17;108:105332. doi: 10.1016/j.ebiom.2024.105332 (PMC11424963; doi:10.1016/j.ebiom.2024.105332)
Supplement: Supplementary Figure and Tables [file mmc1.docx]

**Next generation yellow fever vaccine induces an equivalent immune and transcriptomic profile to the current vaccine: observations from a phase I randomised clinical trial**

Anke Pagnon, PhD,^1^ Christophe Carre, MD,^1^ Marion Aguirre, BSc,^1^ Emilie Chautard, PhD^1^ Sophie Gimenez, UnivDip,^1^ Franck Raynal, MSc,^1^ Emmanuel Feroldi, MD,^1^ Paul Scott, MD,^2^ Kayvon Modjarrad, MD, PhD,^2^ Manuel Vangelisti, MSc,^1^ Nathalie Mantel, PhD^1*^

*^1^Vaccine Research and Development, Sanofi, Marcy l’Etoile, France*

*^2^Emerging Infectious Diseases Branch, Walter Reed Army Institute of Research, Silver Spring, MD, USA*

***Corresponding Author:**

Nathalie Mantel, Vaccine Research and Development, Sanofi, 1541 Avenue Marcel Mérieux, 69280 Marcy l’Etoile, France

Tel:+33(0)4 37 37 93 08; E-mail: nathalie.mantel@sanofi.com

**Supplementary materials**

Table S1. Selected genes and their roles.

| Human Gene Symbol | Associated Blood Transcriptional Module annotations | Role | Ref  ThermoFisher |
| --- | --- | --- | --- |
| AGRN | Unknown | Agrin: critical in the development of the neuromuscular junction. Belongs to protein core of heparan sulfate proteoglycans. Evidence of Agrin expression in APC; Agrin is required for survival and function of monocytic cells. Role for Agrin in T cells and potential importance in immune system regulation^1^ | Hs00394748_m1 |
| APOBEC3A | cell cycle and transcription innate antiviral response | Apolipoprotein B MRNA Editing Enzyme Catalytic Subunit 3A : cytidine deaminase gene family; role in immunity, by restricting transmission of foreign DNA such as viruses Significant impact in vivo on viral genomes of the APOBEC and ADAR deaminases which demonstrably alter viral RNA (and DNA) sequences, often at high frequency. Such deamination events on RNA substrates for APOBEC1, APOBEC3A and ADAR enzymes have also been documented at similar agreed motifs in both unpaired RNA loops and in double stranded RNA secondary structures DEG in DENV3 infected MoDC^2^ | Hs00377444_m1 |
| ATF3 | AP-1 transcription factor network signaling in T cells (I) putative targets of PAX3 enriched in activated dendritic cells (II) Interferon | Activating Transcription Factor 3: activation transcription factor/cAMP responsive element-binding protein family of transcription factors involved in the complex process of cellular stress response. binds the cAMP response element, a sequence present in many viral and cellular promoters. Represses transcription from promoters with ATF sites. | Hs00231069_m1 |
| BATF2 | Interferon | Basic Leucine Zipper ATF-Like Transcription Factor 2: transcription factor that controls the differentiation of lineage-specific cells in the immune system | Hs00912737_m1 |
| BIRC5 | cell cycle and transcription cell cycle (I) C-MYC transcriptional network mitotic cell division | Baculoviral IAP Repeat Containing 5: Multitasking protein that has dual roles in promoting cell proliferation and preventing apoptosis | Hs00977611_g1 |
| BST2 | viral sensing & immunity; IRF2 targets network (I) & (II) Interferon | Bone Marrow Stromal Cell Antigen 2: involved in the growth and development of B-cells. IFN-induced antiviral host restriction factor which efficiently blocks the release of diverse mammalian enveloped viruses by directly tethering nascent virions to the membranes of infected cells. Can stimulate signaling by LILRA4/ILT7 and consequently provide negative feedback to the production of IFN by plasmacytoid dendritic cells in response to viral infection | Hs00171632_m1 |
| C2 | activated dendritic cells complement activation (I) | Part of the classical pathway of the complement system. Activated C1 cleaves C2 into C2a and C2b. The serine proteinase C2a then combines with complement factor 4b to create the C3 or C5 convertase. | Hs00918862_m1 |
| CCL2 | targets of FOSL1/2 AP-1 transcription factor network chemokine cluster (I)& (II) chemokines and receptors enriched for cell migration extracellular region cluster (GO) | C-C Motif Chemokine Ligand 2: chemokine is a member of the CC subfamily which is characterised by two adjacent cysteine residues. This cytokine displays chemotactic activity for monocytes and basophils but not for neutrophils or eosinophils. | Hs00234140_m1 |
| CCR1 | regulation of signal transduction cell cycle and transcription myeloid cell enriched receptors and transporters enriched in monocytes (II) & (III) chemokine cluster (II) CCR1, 7 and cell signaling receptors, cell migration Monocyte surface signature Inflammation | C-C Motif Chemokine Receptor 1: Receptor for a C-C type chemokine. Binds to MIP-1-alpha, MIP-1-delta, RANTES, and MCP-3 and, less efficiently, to MIP-1-beta or MCP-1 and subsequently transduces a signal by increasing the intracellular calcium ions level. Chemokines and their receptors mediated signal transduction are critical for the recruitment of effector immune cells to the site of inflammation. | Hs00174298_m1 |
| CDT1 | cell cycle and transcription cell cycle (I) mitotic cell cycle - DNA replication mitotic cell cycle cell division stimulated CD4+ T cells Cell Cycle | Chromatin Licensing And DNA Replication Factor 1: The protein encoded by this gene is involved in the formation of the pre-replication complex that is necessary for DNA replication. The encoded protein can bind geminin, which prevents replication and may function to prevent this protein from initiating replication at inappropriate origins. | Hs00368864_m1 |
| CMPK2 | Unknown | Cytidine/Uridine Monophosphate Kinase 2: encodes one of the enzymes in the nucleotide synthesis salvage pathway that may participate in terminal differentiation of monocytic cells. CMPK2 is always immediately adjacent to VIPERIN in vertebrate genomes & is co-transcribed with VIPERIN during IFN stimulation, suggesting a functional linkage. CMPK2 primarily functions is to ensure sufficient substrate for VIPERIN-mediated production of ddhCTP during viral infection^3^ | Hs01013364_m1 |
| CXCL10 | innate activation by cytosolic DNA sensing chemokine cluster (I) & (II) RIG-1 like receptor signaling antiviral IFN signature viral sensing & immunity; IRF2 targets network (II) Interferon | C-X-C Motif Chemokine Ligand 10: This antimicrobial gene encodes a chemokine of the CXC subfamily and ligand for the receptor CXCR3. Binding of this protein to CXCR3 results in pleiotropic effects, including stimulation of monocytes, natural killer and T-cell migration, and modulation of adhesion molecule expression | Hs00171042_m1 |
| DDX58 | innate activation by cytosolic DNA sensing activated dendritic cells RIG-1 like receptor signaling antiviral IFN signature viral sensing & immunity; IRF2 targets network (II) Interferon | =RIG-I: Retinoic Acid-Inducible Gene I Viral double-stranded (ds) RNA recognition and the regulation of immune response. RIG-I/MDA5 mediated induction of IFN-a/b pathways.  Known as activated after Dengue, JEV and WNV infections | Hs01061436_m1 |
| DDX60 | activated dendritic cells antiviral IFN signature type I interferon response | DExD/H-Box Helicase 60: Positively regulates DDX58/RIG-I- and IFIH1/MDA5-dependent type I interferon and interferon inducible gene expression in response to viral infection. Binds ssRNA, dsRNA and dsDNA and can promote the binding of DDX58/RIG-I to dsRNA | Hs01102712_m1 |
| DDX60L | Unknown | DExD/H-Box 60 Like: In addition to functions in RNA metabolism, members of this family are involved in anti-viral immunity and act as cytosolic sensors of viral nucleic acids. | Hs01016260_m1 |
| DHX58 | RIG-1 like receptor signaling antiviral IFN signature Interferon | DExH-Box Helicase 58 =LPG2 Involved in RIG-I/MDA5 mediated induction of IFN-alpha/beta pathways & Innate Immune System pathways regulator of DDX58/RIG-I and IFIH1/MDA5 mediated antiviral signaling (both positive and negative depending on interacting virus and target cell). Involved in the innate immune response to various RNA viruses; Can bind both ssRNA & dsRNA, with higher affinity for dsRNA | Hs01597843_m1 |
| DTX3L | Unknown | Deltex E3 Ubiquitin Ligase 3L: E3 ubiquitin-protein ligase which, in association with ADP-ribosyltransferase PARP9, plays a role in DNA damage repair and in interferon-mediated antiviral responses. Positively regulates STAT1-dependent ISG transcription and thus STAT1-mediated control of viral replication. Independently of its catalytic activity, promotes the sorting of chemokine receptor CXCR4 from early endosome to lysosome following CXCL12 stimulation by reducing E3 ligase ITCH activity and thus ITCH-mediated ubiquitination of endosomal sorting complex required for transport ESCRT-0 components HGS and STAM | Hs00370540_m1 |
| EIF2AK2 | innate antiviral response Interferon | Eukaryotic Translation Initiation Factor 2 Alpha Kinase 2 (=PKR): IFN-induced dsRNA-dependent serine/threonine-protein kinase which plays a key role in the innate immune response to viral infection and is also involved in the regulation of signal transduction, apoptosis, cell proliferation and differentiation. Exerts its antiviral activity on a wide range of DNA and RNA viruses including hepatitis C virus (HCV), hepatitis B virus (HBV), measles virus (MV) and herpes simplex virus 1 (HHV-1). Inhibits viral replication via phosphorylation of the alpha subunit of eukaryotic initiation factor 2 (EIF2S1), this phosphorylation impairs the recycling of EIF2S1 between successive rounds of initiation leading to inhibition of translation which eventually results in shutdown of cellular and viral protein synthesis. In addition to serine/threonine-protein kinase activity, also has tyrosine-protein kinase activity and phosphorylates CDK1 at Tyr-4 upon DNA damage, facilitating its ubiquitination and proteosomal degradation. Either as an adapter protein and/or via its kinase activity, can regulate various signaling pathways (p38 MAP kinase, NF-kappa-B and insulin signaling pathways) and transcription factors (JUN, STAT1, STAT3, IRF1, ATF3) involved in the expression of genes encoding proinflammatory cytokines and IFNs. Can regulate NLRP3 inflammasome assembly and the activation of NLRP3, NLRP1, AIM2 and NLRC4 inflammasomes. Can trigger apoptosis via FADD-mediated activation of CASP8 | Hs00169345_m1 |
| EPSTI1 | Interferon | Epithelial Stromal Interaction 1.  EPSTI1 is one of IL-28A-induced ISGs, plays a vital role in IL-28A-mediated antiviral activity against HCV EPSTI1 effectively inhibits HCV replication in the absence of interferon treatment EPSTI1 can activate PKR promoter and induce several PKR-dependent genes, including IFN-β, IFIT1, OAS1, and RNase L | Hs01566789_m1 |
| FPR3 | DC surface signature | Formyl Peptide Receptor 3: powerful neutrophils chemotactic factors; FPRs are involved in antibacterial host defense and inflammation. | Hs01574392_m1 |
| GBP1 | Interferon | Guanylate Binding Protein 1: induced by interferon. Guanylate binding proteins are characterised by their ability to specifically bind guanine nucleotides (GMP, GDP, and GTP) and are distinguished from the GTP-binding proteins by the presence of 2 binding motifs rather than 3. Promote oxidative killing and deliver antimicrobial peptides to autophagolysosomes, providing broad host protection against different pathogen classes. | Hs00977005_m1 |
| GBP3 | Interferon | Guanylate Binding Protein 3: GBPs specifically bind guanine nucleotides (GMP, GDP, and GTP) and contain two of the three consensus motifs found in typical GTP-binding proteins. Exhibits antiviral activity against influenza virus. | Hs00544385_m1 |
| GBP5 | Interferon | Guanylate Binding Protein 5: belongs to the TRAFAC class dynamin-like GTPase superfamily. The encoded protein acts as an activator of NLRP3 inflammasome assembly and has a role in innate immunity and inflammation. As an activator of NLRP3 inflammasome assembly, plays a role in innate immunity and inflammation. Promotes selective NLRP3 inflammasome assembly in response to microbial and soluble, but not crystalline, agents | Hs00369472_m1 |
| HELZ2 | Interferon | Helicase With Zinc Finger 2: belongs to peroxisome proliferator activated receptor alpha interacting complex HELZ2 is an IFN Effector Mediating Suppression of DENV:^4^ IFN antiviral response is mediated by HELZ2 transcriptional upregulation, enrichment of HELZ2 protein levels in nucleus, & activation of a transcriptional program that appears to modulate intracellular lipid state | Hs00375688_m1 |
| HERC5 | antiviral IFN signature type I interferon response enriched in activated dendritic cells (II) Interferon | (HERC5) HECT And RLD Domain Containing E3 Ubiquitin Protein Ligase Family Member 5: Pro-inflammatory cytokines upregulate expression of this gene in endothelial cells. The protein localises to the cytoplasm and perinuclear region and functions as an interferon induced E3 protein ligase that mediates ISGylation of protein targets As USP18 et HERC6, belongs to the ISG15 ubiquitination pathway: Major E3 ligase for ISG15 conjugation. Acts as a positive regulator of innate antiviral response in cells induced by interferon. Functions as part of the ISGylation machinery that recognises target proteins in a broad and relatively non-specific manner. Catalyses ISGylation of IRF3 which results in sustained activation, it attenuates IRF3-PIN1 interaction, which antagonises IRF3 ubiquitination and degradation, and boosts the antiviral response See review^5^ | Hs00180943_m1 |
| HERC6 | Interferon | HECT And RLD Domain Containing E3 Ubiquitin Protein Ligase Family Member 6: related pathways are Class I MHC mediated antigen processing and presentation and Innate Immune System As USP18, belongs to the ISG15 ISGylation pathway^5^ | Hs00215555_m1 |
| IFI16 | Interferon | Interferon Gamma Inducible Protein 16: Binds double-stranded DNA. Binds preferentially to supercoiled DNA and cruciform DNA structures. Seems to be involved in transcriptional regulation. May function as a transcriptional repressor. Could have a role in the regulation of haematopoietic differentiation through activation of unknown target genes. Controls cellular proliferation by modulating the functions of cell cycle regulatory factors including p53/TP53 and the retinoblastoma protein. Involved in innate immune response by recognising viral dsDNA in the cytosol and probably in the nucleus. After binding to viral DNA in the cytoplasm recruits TMEM173/STING and mediates the induction of IFN-beta. Has anti-inflammatory activity and inhibits the activation of the AIM2 inflammasome, probably via association with AIM2. | Hs00986757_m1 |
| IFI27 | enriched in activated dendritic cells (II) Activated (LPS) dendritic cell surface signature | Interferon Alpha Inducible Protein 27: ISG Promotes cell death. Mediates IFN-induced apoptosis characterised by a rapid & robust release of cytochrome C from mitochondria & activation of BAX, caspases 2, 3, 6, 8 and 9 | Hs01086373_g1 |
| IFI35 | viral sensing & immunity; IRF2 targets network (I) Interferon | Interferon Induced Protein 35: Type I Interferon-Dependent Transcript, Upregulates Inflammatory Signaling Pathways by Activating Toll-Like Receptor 3 | Hs00413458_m1 |
| IFI44 | Interferon | Interferon Induced Protein 44: Interferon Stimulated gene This protein aggregates to form microtubular structures | Hs00197427_m1 |
| IFI44L | Interferon | Interferon Induced Protein 44 Like: Interferon Stimulated gene | Hs00915292_m1 |
| IFI6 | Activated (LPS) dendritic cell surface signature | IFN Alpha Inducible Protein 6. plays a critical role in the regulation of apoptosis. | Hs00242571_m1 |
| IFIH1 | activated dendritic cells RIG-1 like receptor signaling antiviral IFN signature type I interferon response enriched in activated dendritic cells (II) Interferon | Interferon Induced With Helicase C Domain 1 (=MDA5: Melanoma Differentiation-Associated Protein 5): Innate immune receptor = cytoplasmic sensor of viral nucleic acids that plays a major role in sensing viral infection & in the activation of a cascade of antiviral responses including the induction of type I IFN and proinflammatory cytokines. Its ligands include mRNA lacking 2-O-methylation at 5’ cap & long-dsRNA (>1 kb). Upon ligand binding it associates with mitochondria antiviral signaling protein (MAVS/IPS1) which activates the IKK-related kinases: TBK1 and IKBKE which phosphorylate interferon regulatory factors: IRF3 and IRF7 which in turn activate transcription of antiviral immunological genes, including type I IFN. | Hs00223420_m1 |
| IFIT1 | antiviral IFN signature type I interferon response innate antiviral response enriched in activated dendritic cells (II) Interferon | Interferon-induced protein with tetratricopeptide repeats 1B: IFIT1B = IFIT1: This gene encodes a protein containing tetratricopeptide repeats that was originally identified as induced upon treatment with interferon. The encoded protein may inhibit viral replication and translational initiation. Interferon-induced antiviral RNA-binding protein that specifically binds single-stranded RNA bearing a 5-triphosphate group (PPP-RNA), thereby acting as a sensor of viral single-stranded RNAs and inhibiting expression of viral messenger RNAs. | Hs03027069_s1 |
| IFIT2 | viral sensing & immunity; IRF2 targets network (II) enriched in activated dendritic cells (II) Interferon | Interferon-induced protein with tetratricopeptide repeats 2 IFN-induced antiviral protein which inhibits expression of viral messenger RNAs lacking 2-O-methylation of the 5 cap. Can promote apoptosis | Hs00533665_m1 |
| IFIT3 | enriched in activated dendritic cells (II) Interferon | Interferon-induced protein with tetratricopeptide repeats 3 IFN-induced antiviral protein which acts as an inhibitor of cellular as well as viral processes: cell migration, proliferation, signaling, and viral replication. Enhances MAVS-mediated host antiviral responses by serving as an adapter bridging TBK1 to MAVS which leads to activation of TBK1 and phosphorylation of IRF3. Phosphorylated IRF3 translocates into nucleus to promote antiviral gene transcription. Exhibits an antiproliferative activity via the up regulation of cell cycle negative regulators CDKN1A/p21 & CDKN1B/p27. Can negatively regulate the apoptotic effects of IFIT2. | Hs01922752_s1 |
| IFIT5 | Interferon | Interferon Induced Protein with Tetratricopeptide Repeats 5: broad and adaptable RNA structure recognition important for RNA recognition specificity in human antiviral innate immune response; acting as a sensor of viral single-stranded RNAs | Hs00202721_m1 |
| IFITM1 | type I interferon response Interferon | Interferon Induced Transmembrane Protein 1: IFN-induced antiviral protein which inhibits the entry of viruses to the host cell cytoplasm, permitting endocytosis, but preventing subsequent viral fusion and release of viral contents into the cytosol. Active against Dengue, ZIKV and WNV | Hs01652522_g1 |
| IRF7 | innate activation by cytosolic DNA sensing activated dendritic cells RIG-1 like receptor signaling antiviral IFN signature type I interferon response innate antiviral response Interferon | Interferon Regulatory Factor 7: member of the interferon regulatory transcription factor family. IRF7 has been shown to play a role in the transcriptional activation of virus-inducible cellular genes, including interferon beta chain genes. Inducible expression of IRF7 is largely restricted to lymphoid tissue | Hs00185375_m1 |
| ISG15 | RIG-1 like receptor signaling Interferon | ISG15 Ubiquitin-Like Modifier: activated by IFNa & -b. Several functions described: chemotactic activity towards neutrophils, direction of ligated target proteins to intermediate filaments, cell-to-cell signaling, & antiviral activity. Key role in the innate immune response to viral infection either via its conjugation to a target protein (ISGylation) or via its action as a free or unconjugated protein. ISGylation involves a cascade of enzymatic reactions involving E1, E2, and E3 enzymes which catalyse the conjugation of ISG15 to a lysine residue in the target protein. Target proteins : IFIT1, MX1/MxA, PPM1B, UBE2L6, UBA7, CHMP5, CHMP2A, CHMP4B and CHMP6, EIF2AK2/PKR which results in its activation, DDX58/RIG-I which inhibits its function in antiviral signaling response, EIF4E2 which enhances its cap structure-binding activity and translation-inhibition activity, UBE2N & UBE2E1 which negatively regulates their activity, IRF3 which inhibits its ubiquitination & degradation & FLNB which prevents its ability to interact with the upstream activators of the JNK cascade thereby inhibiting IFNA-induced JNK signaling. See review^5^ | Hs00192713_m1 |
| JUP | regulation of signal transduction immune activation - generic cluster transcriptional targets of glucocorticoid receptor collagen, TGFB family et al cell adhesion, membrane cell cell adhesion | Junction Plakoglobin: constituent of sub-membranous plaques of both desmosomes and intermediate junctions | Hs00158408_m1 |
| KCTD14 | Unknown | Potassium Channel Tetramerisation Domain Containing 14: unknown function^6^  related pathways: Hepatic ABC Transporters & Activation of cAMP-Dependent PKA Ubiquitin ligand –antiviral role in Dengue & EV71 infections DEG in transcriptomics analysis DHF vs DF (upregulated in DHF) | Hs01928822_s1 |
| KIAA0101 | cell cycle and transcription mitotic cell cycle in stimulated CD4 T cells E2F transcription factor network E2F1 targets (Q3) cell division stimulated CD4+ T cells Cell Cycle | = PCNA Clamp Associated Factor: PCNA-binding protein that acts as a regulator of DNA repair during DNA replication. Following DNA damage, the interaction with PCNA is disrupted, facilitating the interaction between monoubiquitinated PCNA and the translesion DNA synthesis DNA polymerase eta (POLH) at stalled replisomes, facilitating the bypass of replication-fork-blocking lesions. Also acts as a regulator of centrosome number. | Hs00207134_m1 |
| KLHDC7B | Unknown | Kelch Domain Containing 7B : unknown function Unclear role / DEG HCV infection^7^ | Hs00536653_s1 |
| LAMP3 | enriched in activated dendritic cells (II) Interferon | Lysosomal Associated Membrane Protein 3: ISG; May play a role in dendritic cell function and in adaptive immunity. | Hs01111316_m1 |
| LY6E | Interferon | Lymphocyte Antigen 6 Family Member E: Involved in T-cell development; In vitro involved in virus entry^8^ | Hs00158942_m1 |
| MX1 | Interferon | MX Dynamin Like GTPase 1: This gene encodes a guanosine triphosphate (GTP)-metabolizing protein that participates in the cellular antiviral response. Interferon-induced dynamin-like GTPase with antiviral activity against a wide range of RNA viruses and some DNA viruses | Hs00895608_m1 |
| MX2 | enriched in activated dendritic cells (II) | MX Dynamin Like GTPase 2: induced by type 1 IFN, inhibit viral replication. | Hs01550808_m1 |
| MZB1 | Unknown | Marginal Zone B & B1 Cell Specific Protein: associates with IgM heavy & light chains and promotes IgM assembly and secretion. May exert its effect by acting as a molecular chaperone or as an oxidoreductase as it displays a low level of oxidoreductase activity. Isoform 2 may be involved in regulation of apoptosis. Helps to diversify peripheral B-cell functions by regulating Ca(2+) stores, antibody secretion and integrin activation. Also act as a hormone-regulated adipokine/proinflammatory cytokine that is implicated in causing chronic inflammation, affecting cellular expansion and blunting insulin response in adipocytes. May have a role in the onset of insulin resistance. | Hs00414907_m1 |
| OAS1 | antiviral IFN signature innate antiviral response Interferon | 2'-5'-Oligoadenylate Synthetase 1: activates latent RNase L, which results in cellular & viral RNA degradation & resulting in the inhibition of protein synthesis, thus terminating viral replication Also participate to detection of foreign RNA | Hs00973637_m1 |
| OAS2 | Interferon | 2'-5'-Oligoadenylate Synthetase 2: This gene encodes a member of the 2-5A synthetase family, essential proteins involved in the innate immune response to viral infection. The encoded protein is induced by interferons and uses adenosine triphosphate in 2'-specific nucleotidyl transfer reactions to synthesise 2',5'-oligoadenylates (2-5As). These molecules activate latent RNase L, which results in viral RNA degradation and the inhibition of viral replication. Interferon-induced, dsRNA-activated antiviral enzyme which plays a critical role in cellular innate antiviral response | Hs00942643_m1 |
| OAS3 | antiviral IFN signature innate antiviral response Interferon | 2'-5'-Oligoadenylate Synthetase 3: IFN-induced, dsRNA-activated antiviral enzyme which plays a critical role in cellular innate antiviral response. In addition, it may also play a role in other cellular processes such as apoptosis, cell growth, differentiation and gene regulation. Synthesises preferentially dimers of 2-5-oligoadenylates (2-5A) from ATP which then bind to the inactive monomeric form of ribonuclease L (RNase L) leading to its dimerisation and subsequent activation. Activation of RNase L leads to degradation of cellular as well as viral RNA, resulting in the inhibition of protein synthesis, thus terminating viral replication. Can mediate the antiviral effect via the classical RNase L-dependent pathway or an alternative antiviral pathway independent of RNase L. | Hs00196324_m1 |
| OASL | innate antiviral response Interferon | 2'-5'-Oligoadenylate Synthetase Like: double-stranded RNA binding protein involved in innate immune system and interferon gamma signaling. | Hs00984387_m1 |
| PARP10 | Interferon | Poly-(ADP-Ribose) Polymerase Family Member 10: regulate gene transcription by altering chromatin organisation by adding ADP-ribose to histones. PARPs can also function as transcriptional cofactors. ADP-ribosyltransferase that mediates mono-ADP-ribosylation of glutamate and aspartate residues on target proteins | Hs00361105_m1 |
| PARP12 | Interferon | Poly-(ADP-Ribose) Polymerase Family Member 12: catalyses post-translational modification of proteins by addition of multiple ADP-ribose moieties; = ISG Canonical Pathway: Death Receptor Signaling; Retinoic acid Mediated Apoptosis Signaling  Regulated by IFNA2, IFNB1, SIRT1, IFNAR1, CNOT7, MAVS, IRF5, IRF7, IRF3, myeloid DC, HCV JFH-1, EIF2AK2, NKX2-3, TRIM24 Several studies demonstrated the involvement of PARPs in the antiviral response is widespread, with regulation of both viral & antiviral defense transcripts PARP-7, -10, & the long isoform of PARP-12 function as important & very potent regulators of cellular translation & virus replication^9^ PARP-12 suppresses ZIKV infection through PARP-dependent degradation of NS1 & NS3^10^. | Hs01104912_m1 |
| PARP14 | Interferon | Poly-(ADP-Ribose) Polymerase Family Member 14: ADP-ribosyltransferase that mediates mono-ADP-ribosylation of glutamate residues on target proteins. The encoded anti-apoptotic protein may regulate aerobic glycolysis and promote survival of cancer cells. | Hs00393816_m1 |
| PARP9 | type I interferon response enriched in activated dendritic cells (II) Interferon | Poly-(ADP-Ribose) Polymerase Family Member 9: catalyses the post-translational modification of proteins by the addition of multiple ADP-ribose moieties. PARP transfers ADP-ribose from nicotinamide dinucleotide (NAD) to Glu/Asp residues on the substrate protein. plays a role in DNA damage repair and in immune responses including interferon-mediated antiviral defenses. Dispensable for BCR assembly through V(D)J recombination and class switch recombination. In macrophages, positively regulates pro-inflammatory cytokines production in response to IFNg stimulation by suppressing PARP14-mediated STAT1 ADP-ribosylation and thus promoting STAT1 phosphorylation. | Hs00967084_m1 |
| PLSCR1 | activated dendritic cells type I interferon response innate antiviral response Interferon | Phospholipid Scramblase 1:  May mediate accelerated ATP-independent bidirectional trans-bilayer migration of phospholipids upon binding calcium ions that results in a loss of phospholipid asymmetry in the plasma membrane. May play a central role in the initiation of fibrin clot formation, in the activation of mast cells and in the recognition of apoptotic and injured cells by the reticuloendothelial system.  May play a role in the antiviral response of interferon (IFN) by amplifying and enhancing the IFN response through increased expression of select subset of potent antiviral genes. May contribute to cytokine-regulated cell proliferation and differentiation. | Hs01062171_m1 |
| PML | antiviral IFN signature Interferon | Promyelocytic Leukemia: TRIM family transcription factor. Functions via its association with PML-nuclear bodies (PML-NBs) in a wide range of important cellular processes, including tumor suppression, transcriptional regulation, apoptosis, senescence, DNA damage response, and viral defense mechanisms.  The nuclear isoforms (isoform PML-1, isoform PML-2, isoform PML-3, isoform PML-4 and isoform PML-5) in concert with SATB1 are involved in local chromatin-loop remodeling and gene expression regulation at the MHC-I locus. Isoform PML-2 is required for efficient IFN-gamma induced MHC II gene transcription via regulation of CIITA. Cytoplasmic PML is involved in the regulation of the TGF-beta signaling pathway. PML also regulates transcription activity of ELF4 and can act as an important mediator for TNF-alpha- and IFN-alpha-mediated inhibition of endothelial cell network formation and migration. Exhibits antiviral activity against both DNA and RNA viruses. The antiviral activity can involve one or several isoform(s) and can be enhanced by the permanent PML-NB-associated protein DAXX or by the recruitment of p53/TP53 within these structures | Hs00971694_m1 |
| RSAD2 | activated dendritic cells antiviral IFN signature type I interferon response innate antiviral response enriched in activated dendritic cells (II) Interferon | Radical S-Adenosyl Methionine Domain Containing 2 (VIPERIN= Virus inhibitory protein, Endoplasmic Reticulum-associated, Interferon inducible) Interferon-inducible iron-sulfur (4FE-4S) cluster-binding antiviral protein which plays a major role in the cell antiviral state induced by type I & type II IFN. Can inhibit a wide range of DNA and RNA viruses, including HCMV, HCV, WNV, DENV, VSV, & HIV-1.  Promotes TLR7 and TLR9-dependent production of IFN-beta production in plasmacytoid dendritic cells (pDCs) & Plays a role in CD4+ T-cells activation and differentiation.^11^ | Hs00369813_m1 |
| SAMD4A | Undetermined | Sterile Alpha Motif Domain Containing 4A: RNA-binding domain that functions as a posttranscriptional regulator by binding to an RNA sequence motif  Stress granule protein | Hs00324455_m1 |
| SAMD9L | Interferon | Sterile Alpha Motif Domain Containing 9 Like: cytoplasmic protein that acts as a tumor suppressor but also plays a key role in cell proliferation and the innate immune response to viral infection. May be involved in endosome fusion. Mediates down-regulation of growth factor signaling via internalisation of growth factor receptors. | Hs00416109_m1 |
| SERPING1 | cell activation (IL15, IL23, TNF) activated dendritic cells antiviral IFN signature complement activation (I) enriched in activated dendritic cells (II) Interferon | Serpin Family G Member 1: highly glycosylated plasma protein involved in the regulation of the complement cascade. Its protein inhibits activated C1r and C1s of the first complement component and thus regulates complement activation. May play a potentially crucial role in regulating important physiological pathways including complement activation, blood coagulation, fibrinolysis and the generation of kinins. | Hs00163781_m1 |
| SIGLEC1 | enriched in activated dendritic cells (II) DC surface signature Activated (LPS) dendritic cell surface signature | Sialic Acid Binding Ig Like Lectin 1 (=CD169): type I transmembrane protein expressed only by subpopulation of macrophages & mediator of cell-cell interactions. Involved in pathways Immuno-regulatory interactions between Lymphoid / non-Lymphoid cell & Innate Immune System CD169, was originally reported as a marker of one macrophage subpopulation isolated from BM, LN, liver, & spleen. These cells had the ability to bind red cells. The biological functions of CD169+ macrophages are still imprecise. CD169+ macrophages do not mediate phagocytosis and are mainly involved in the regulation of the immune system rather than in steady-state haematopoiesis. It has been proposed some activity under viral infection Up-regulated after MoDC with DENV3 & CYD3 ^2^ upregulated in ≠ transcriptomic analysis after infection with Flavivirus but never discussed Siglec1 suppresses antiviral innate immune response by inducing TBK1 degradation via the ubiquitin ligase TRIM27^12^ DEG after JEV infection^13^_ DENV traffics to and replicates in both CD169+ & SIGN-R1+ macrophages of the splenic marginal zone or draining LN^14^ | Hs00224991_m1 |
| SLFN13 | Unknown | Schlafen Family Member 13: Endoribonuclease that cleaves tRNAs and rRNAs  Although the effects of Schlafen Family genes (slfns) are still poorly understood, it has been confirmed that slfns are involved in the defense of immune system and regulate immune cells proliferation and differentiation; the slfn proteins also disturb replication and virulence of viruses (demonstrated with HIV and camelpox)^15^ Slfn expression can be induced by type I interferon & ISG^16^ | Hs00431187_m1 |
| SPATS2L | Unknown | Spermatogenesis Associated Serine Rich 2 Like = DNAPTP6 DEG in other transcriptomics analysis after Flavivirus infection,^17^ but not commented | Hs01016364_m1 |
| STAT1 | type I interferon response Interferon | (STAT1) Signal transducer and transcription activator that mediates cellular responses to interferons (IFNs). Phosphorylated by the receptor associated kinases, and then form homo- or heterodimers that translocate to the cell nucleus where they act as transcription activators. This protein can be activated by various ligands including interferon-alpha, interferon-gamma, EGF, PDGF and IL6. This protein mediates the expression of a variety of genes, which is thought to be important for cell viability in response to different cell stimuli and pathogens | Hs01013996_m1 |
| STAT2 | Interferon | Signal Transducer and Activator of Transcription 2: Signal transducer and activator of transcription that mediates signaling by type I IFNs. Following type I IFN binding to cell surface receptors, Jak kinases (TYK2 and JAK1) are activated, leading to tyrosine phosphorylation of STAT1 and STAT2. The phosphorylated STATs dimerise, associate with IRF9/ISGF3G to form a complex termed ISGF3 transcription factor, that enters the nucleus. ISGF3 binds to the IFN stimulated response element (ISRE) to activate the transcription of interferon stimulated genes, which drive the cell in an antiviral state | Hs01013123_m1 |
| TLR3 | Unknown | Toll like receptor 3: plays a fundamental role in pathogen recognition & activation of innate immunity, activated by double-stranded RNA | Hs00152933_m1 |
| TLR7 | regulation of antigen presentation and immune response TLR and inflammatory signaling immune activation - generic cluster endoplasmic reticulum enriched in myeloid cells and monocytes viral sensing & immunity; IRF2 targets network (I) MHC-TLR7-TLR8 cluster | Toll Like Receptor 7: plays a fundamental role in pathogen recognition & activation of innate immunity, activated by single-stranded RNA | Hs00152971_m1 |
| TNFRSF17 | Plasma cells & B cells, immunoglobulins plasma cells, immunoglobulins Memory B cell surface signature Plasma Cells | TNF Receptor Superfamily Member 17: receptor is preferentially expressed in mature B lymphocytes and may be important for B cell development and autoimmune response. This receptor has been shown to specifically bind to the tumor necrosis factor (ligand) superfamily, member 13b (TNFSF13B/TALL-1/BAFF), and to lead to NF-kappa B and MAPK8/JNK activation. This receptor also binds to various TRAF family members, and thus may transduce signals for cell survival and proliferation. Promotes B-cell survival and plays a role in the regulation of humoral immunity. | Hs00171292_m1 |
| TNFSF13B | TLR8-BAFF network viral sensing & immunity; IRF2 targets network (I) & (II) enriched in monocytes (IV) & (surface) Activated (LPS) dendritic cell surface signature Inflammation | TNF Superfamily Member 13b: cytokine, belongs to the TNF ligand family; is expressed in B cell lineage cells, and acts as a potent B cell activator. Play an important role in the proliferation and differentiation of B cells, | Hs00198106_m1 |
| TOR1B | Unknown | Torsin Family 1 Member B: ATPase found primarily in RE & nuclear envelope; chaperone & play a role in maintaining the integrity of the nuclear envelope and endoplasmic reticulum. Involved in clathrin-mediated endocytosis and Vesicle-mediated transport pathways. Associated with autophagosome in HUH7.5 cells | Hs00979020_m1 |
| TRIM22 | Interferon | Tripartite Motif Containing 22: Interferon-induced antiviral protein involved in cell innate immunity. The antiviral activity could in part be mediated by TRIM22-dependent ubiquitination of viral proteins. | Hs01001179_m1 |
| UBE2L6 | Interferon | Member of the E2 ubiquitin-conjugating enzyme family – Involved in Class I MHC mediated antigen processing and presentation. Catalyses the covalent attachment of ubiquitin or ISG15 to other proteins | Hs01125548_m1 |
| UNC93B1 | endoplasmic reticulum T cell activation (IV) Interferon | Unc-93 Homolog B1, TLR Signaling Regulator: Plays an important role in innate and adaptive immunity by regulating nucleotide-sensing Toll-like receptor (TLR) signaling. Required for the transport of a subset of TLRs (including TLR3, TLR7 and TLR9) from the endoplasmic reticulum to endo-lysosomes where they can engage pathogen nucleotides and activate signaling cascades. May play a role in autoreactive B-cells removal. | Hs00276771_m1 |
| USP18 | viral sensing & immunity; IRF2 targets network (I) & (II) type I interferon response | Ubiquitin Specific Peptidase 18: highly expressed in liver & thymus; efficiently cleaves only ISG15 fusions, probably function in regulating IFN responses USP18-Based Negative Feedback Control Is Induced by Type I and Type III IFN & Specifically Inactivates IFNα Response^18^ ZIKV constitutively induced ISG15, HERC5, & USP18, which are linked to HCV persistence & IFN regulation, chemokine CCL5, which is associated with immunopathogenesis, as well as cell survival factors^19^ | Hs00276441_m1 |
| XAF1 | Interferon | XIAP Associated Factor 1: Seems to function as a negative regulator of members of the IAP (inhibitor of apoptosis protein) family. Inhibits anti-caspase activity of BIRC4. Induces cleavage and inactivation of BIRC4 independent of caspase activation. Mediates TNF-alpha-induced apoptosis and is involved in apoptosis | Hs01550142_m1 |
| ZBP1 | innate activation by cytosolic DNA sensing viral sensing & immunity; IRF2 targets network (I) & (II) Interferon | Z-DNA Binding Protein 1: Participates in the detection by the hosts innate immune system of DNA from viral, bacterial or even host origin. Acts as a cytoplasmic DNA sensor which, when activated, induces the recruitment of TBK1 and IRF3 to its C-terminal region and activates the downstream interferon regulatory factor (IRF) and NF-kappa B transcription factors, leading to type-I interferon production | Hs00229199_m1 |
| ZCCHC2 | Unknown | Zinc Finger protein CCHC-Type Containing 2 Host factor and interferon (IFN)-stimulated gene (ISG) product, zinc-finger antiviral protein (ZAP), inhibits a number of diverse viruses by usurping and intersecting with multiple cellular pathways. Infection of JEV but not YFV, DENV or ZIKV was blocked by ZAP overexpression, and depletion of endogenous ZAP enhanced JEV replication^20^ | Hs00214742_m1 |
| IGHG1 | Unknown | immunoglobulin heavy constant gamma 1 | Custom assay |
| IGHG2 | Unknown | immunoglobulin heavy constant gamma 2 | Custom assay |
| IGHG3 | Unknown | immunoglobulin heavy constant gamma 3 | Custom assay |
| IGHG4 | Unknown | immunoglobulin heavy constant gamma 4 | Custom assay |
| IGHM | Unknown | immunoglobulin heavy constant mu | Custom assay |

Table S2. Serum cytokine/chemokine levels following vaccination with vYF (4, 5 and 6 Log CCID_50_) or YF-VAX (FAS).

| Cytokine/chemokine | Group | Parameter | V01(D0) | V02(D1) | V03(D3) | V04(D5) | V05(D7) | V06(D10) | V07(D14) |
| --- | --- | --- | --- | --- | --- | --- | --- | --- | --- |
| Chemokine (C-C Motif) Ligand 5 (pg/mL) | YF-VAX | N | 18 | 18 | 13 | 12 | 18 | 18 | 18 |
|  |  | GM | 118,328 | 119,357 | 102,198 | 119,505 | 111,265 | 116,078 | 139,594 |
|  |  | 95% CI | [98,138;142,671] | [101,640;140,161] | [73,602;141,906] | [93,271;153,119] | [85,260;145,201] | [91,975;146,498.0] | [101,449;192,082] |
|  | vYF 4 Log | N | 18 | 18 | 13 | 13 | 16 | 17 | 18 |
|  |  | GM | 198,437 | 193,658 | 204,295 | 186,452 | 166,640 | 184,457 | 184,048 |
|  |  | 95% CI | [134,561;292,633] | [130,566.0;287,237] | [137,055;304,522] | [119,177;291,703] | [112,164;247,575] | [126,845;268,234] | [130,064;260,438] |
|  | vYF 5 Log | N | 18 | 18 | 13 | 12 | 18 | 17 | 18 |
|  |  | GM | 176,706 | 178,802 | 174,985 | 144,692 | 151,201 | 192,678 | 180,686 |
|  |  | 95% CI | [119,783;260,678] | [126,687;252,357] | [111,002;275,849] | [96,357;217,272] | [102,662;222,691] | [149,337;248,597] | [120,625;270,654] |
|  | vYF 6 Log | N | 18 | 17 | 11 | 11 | 18 | 16 | 18 |
|  |  | GM | 153,972 | 149,810 | 142,044 | 168,334 | 155,475 | 149,432 | 179,066 |
|  |  | 95% CI | [120,748;196,336] | [120,123;186,835] | [90,805;222,194] | [115,410;245,527] | [111,478;216,837] | [103,664;215,406] | [130,425;245,848] |
| Chemokine (C-X-C Motif) Ligand 10 (pg/mL) | YF-VAX | N | 18 | 18 | 13 | 12 | 18 | 18 | 18 |
|  |  | GM | 126 | 168 | 171 | 226 | 266 | 169 | 132 |
|  |  | 95% CI | [78.5;201.7] | [99.5;282.3] | [125.0;234.91 | [111.9;455.6] | [182.7;386.0] | [124.8;228.1] | [99.0;175.8] |
|  | vYF 4 Log | N | 18 | 18 | 13 | 13 | 16 | 17 | 18 |
|  |  | GM | 83.2 | 91.3 | 121.0 | 106.9 | 178.6 | 129.6 | 98.9 |
|  |  | 95% CI | [57.5;120.3] | [59.2;140.8] | [69.8;209.7] | [56.4;202.5] | [107.4;296.8] | [85.2;197.1] | [64.5;151.7] |
|  | vYF 5 Log | N | 18 | 18 | 13 | 12 | 18 | 17 | 18 |
|  |  | GM | 87.2 | 88.7 | 128.6 | 118.9 | 184.0 | 149.2 | 109.2 |
|  |  | 95% CI | [62.4;121.7] | [66.1;119.0] | [94.2;175.7] | [71.5;197.7] | [133.1;254.5] | [119.6;186.2] | [84.9;140.6] |
|  | vYF 6 Log | N | 18 | 17 | 11 | 11 | 18 | 16 | 18 |
|  |  | GM | 109.1 | 122.1 | 183.9 | 144.9 | 220.3 | 154.9 | 113.6 |
|  |  | 95% CI | [72.3;164.7] | [83.9;177.6] | [123.3;274.2] | [89.7;234.2] | [149.6;324.6] | [115.4;208.0] | [88.4;146.0] |
| Granulocyte Colony Stimulating Factor (pg/mL) | YF-VAX | N | 18 | 18 | 13 | 12 | 18 | 18 | 18 |
|  |  | GM | 14 | 18 | 18 | 26 | 21 | 23 | 18 |
|  |  | 95% CI | [5.4;34.6] | [7.1;46.0] | [6.5;50.9] | [8.0;84.5] | [8.5;53.9] | [9.3;55.5] | [7.0;46.2] |
|  | vYF 4 Log | N | 18 | 18 | 13 | 13 | 16 | 17 | 18 |
|  |  | GM | 7.2 | 7.3 | 9.2 | 9.8 | 7.0 | 5.7 | 5.8 |
|  |  | 95% CI | [3.7;14.2] | [3.7;14.4] | [3.9;21.3] | [3.3;29.1] | [3.4;14.6] | [3.2;10.4] | [3.3;10.4] |
|  | vYF 5 Log | N | 18 | 18 | 13 | 12 | 18 | 17 | 18 |
|  |  | GM | 6.9 | 5.4 | 5.1 | 6.3 | 5.3 | 5.5 | 5.9 |
|  |  | 95% CI | [3.0;15.6] | [2.8;10.4] | [2.3;11.1] | [2.3;17.0] | [2.7;10.5] | [2.7;11.1] | [3.0;11.6] |
|  | vYF 6 Log | N | 18 | 17 | 11 | 11 | 18 | 16 | 18 |
|  |  | GM | 18.3 | 18.7 | 21.9 | 35.7 | 18. 9 | 17.6 | 21.4 |
|  |  | 95% CI | [8.0;41. 8] | [7.9;44.5] | [6.5;74.0] | [12.0;105. 7] | [8.1;44.3] | [6.9;44.5] | [9.3;49.4] |
| Interferon Gamma (pg/mL) | YF-VAX | N | 18 | 18 | 13 | 12 | 18 | 18 | 18 |
|  |  | GM | 1 | 1 | 2 | 2 | 2 | 1 | 1 |
|  |  | 95% CI | [0.8;2.5] | [0.9;2.6] | [0.7;3.6] | [0.8;3.0] | [0.8;3.4] | [0.8;2.6] | [0.7;2.1] |
|  | vYF 4 Log | N | 18 | 18 | 13 | 13 | 16 | 17 | 18 |
|  |  | GM | 1.0 | 1.0 | 1.0 | 1.1 | 1.3 | 1.4 | 1.0 |
|  |  | 95% CI | [0.7;1.6] | [0.7;1.6] | [0.6;1.5] | [0.6;1.9] | [0.8;2.1] | [0.9;2.2] | [0.7;1.4] |
|  | vYF 5 Log | N | 18 | 18 | 13 | 12 | 18 | 17 | 18 |
|  |  | GM | 1.0 | 1.1 | 0.8 | 1.5 | 1.54 | 1.4 | 1.2 |
|  |  | 95% CI | [0.6;1.7] | [0.7;1.7] | [0.7;1.0] | [0.7;3.2] | [0.9;2.7] | [0.8;2.4] | [0.7;2.0] |
|  | vYF 6 Log | N | 18 | 17 | 11 | 11 | 18 | 16 | 18 |
|  |  | GM | 2.0 | 1.8 | 2.3 | 3.0 | 2.9 | 3.0 | 2.3 |
|  |  | 95% CI | [1.2;3.5] | [1.0;3.2] | [1.0;5.7] | [1.3;6.5] | [1.6;5.2] | [1.5;5.7] | [1.2;4.2] |
| Interleukin 1 Alpha (pg/mL) | YF-VAX | N | 18 | 18 | 13 | 12 | 18 | 18 | 18 |
|  |  | GM | 4 | 4 | 6 | 7 | 5 | 4 | 5 |
|  |  | 95% CI | [1.9;7. 8] | [2.0;8.7] | [1.7;19.3] | [3.4;12.7] | [1.9;11.5] | [2.1;8.5] | [2.6;9.6] |
|  | vYF 4 Log | N | 18 | 18 | 13 | 13 | 16 | 17 | 18 |
|  |  | GM | 4.0 | 3.5 | 3.7 | 3.6 | 4.2 | 3.5 | 3.2 |
|  |  | 95% CI | [2.2;7.4] | [2.0;6.3] | [1.9;7.3] | [1.7;7.9] | [2.2;8.2] | [2.0;5.9] | [1.8;5.7] |
|  | vYF 5 Log | N | 18 | 18 | 13 | 12 | 18 | 17 | 18 |
|  |  | GM | 4.3 | 3.9 | 3.2 | 4.0 | 3.8 | 3.9 | 3.7 |
|  |  | 95% CI | [2.2;8.4] | [2.1;7.3] | [1.9;5.3] | [1.6;9.8] | [2.0;7.4] | [1.9;7.9] | [1.9;7.5] |
|  | vYF 6 Log | N | 18 | 17 | 11 | 11 | 18 | 16 | 18 |
|  |  | GM | 6.89 | 6.8 | 7.9 | 16.3 | 8.3 | 6.7 | 7.8 |
|  |  | 95% CI | [3.3;14.0] | [3.3;14.2] | [3.0;20.4] | [6.4;41.5] | [3.6;19.0] | [3.3;13.5] | [3.5;17.5] |
| Interleukin 1 Beta (pg/mL) | YF-VAX | N | 18 | 18 | 13 | 12 | 18 | 18 | 18 |
|  |  | GM | 3 | 3 | 1 | 5 | 3 | 3 | 3 |
|  |  | 95% CI | [1.1;8.0] | [1.0;7.6] | [0.4;5.4] | [1.6;17.2] | [1.1;8.5] | [1.0;7.4] | [1.1;8.5] |
|  | vYF 4 Log | N | 18 | 18 | 13 | 13 | 16 | 17 | 18 |
|  |  | GM | 1.7 | 1.7 | 2.1 | 2.3 | 2.1 | 1.6 | 1.7 |
|  |  | 95% CI | [0.6;4.7] | [0.6;4.8] | [0.6;7.0] | [0.6;8.6] | [0.7;6.2] | [0.6;4.4] | [0.7;4.5] |
|  | vYF 5 Log | N | 18 | 18 | 13 | 12 | 18 | 17 | 18 |
|  |  | GM | 3.3 | 2.5 | 2.2 | 3.8 | 3.3 | 2.6 | 2.9 |
|  |  | 95% CI | [1.2;9.0] | [0.9;7.0] | [0.8;6.0] | [0.9;16.0] | [1.2;9.4] | [0.9;7.6] | [1.0;8.3] |
|  | vYF 6 Log | N | 18 | 17 | 11 | 11 | 18 | 16 | 18 |
|  |  | GM | 4.8 | 4.9 | 3.2 | 17.8 | 6.0 | 6.2 | 6.1 |
|  |  | 95% CI | [1.6;14. 9] | [1.5;15.7] | [0.6;16.4] | [6.4;49. 7] | [2.0;17.5] | [1.9;20.4] | [1.9;19.8] |
| Interleukin 10 (pg/mL) | YF-VAX | N | 18 | 18 | 13 | 12 | 18 | 18 | 18 |
|  |  | GM | 7 | 7 | 6 | 11 | 8 | 8 | 7 |
|  |  | 95% CI | [2.5;18.6] | [2.4;19.2] | [1.8;20.6] | [2.5;47.5] | [2.8;23.8] | [2.9;22.2] | [2.2;19.5] |
|  | vYF 4 Log | N | 18 | 18 | 13 | 13 | 16 | 17 | 18 |
|  |  | GM | 1.8 | 1.5 | 1.9 | 2.3 | 2.1 | 1.6 | 1.5 |
|  |  | 95% CI | [1.0;3.2] | [0.9;2.7] | [0.8;4.1] | [0.7;7.5] | [1.1;4.1] | [0.9;2.9] | [0.9;2.7] |
|  | vYF 5 Log | N | 18 | 18 | 13 | 12 | 18 | 17 | 18 |
|  |  | GM | 3.0 | 2.2 | 2.4 | 2.3 | 2.3 | 2.2 | 2.1 |
|  |  | 95% CI | [1. 2;7.5] | [1.0;4.5] | [0.9;6.2] | [0.8;6.6] | [1.0;5.0] | [1.0;4.9] | [1.0;4.4] |
|  | vYF 6 Log | N | 18 | 17 | 11 | 11 | 18 | 16 | 18 |
|  |  | GM | 8.1 | 9.0 | 12.2 | 20.1 | 8.2 | 8.1 | 8 |
|  |  | 95% CI | [2.9;22.4] | [3.2;25.7] | [2.6;56.5] | [5.5;73.4] | [2.8;24.1] | [2.7;24.5] | [2.9;21.9] |
| Interleukin 12 p40 (pg/mL) | YF-VAX | N | 18 | 18 | 13 | 12 | 18 | 18 | 18 |
|  |  | GM | 24 | 24 | 26 | 23 | 31 | 26 | 26 |
|  |  | 95% CI | [15.8;37.0] | [15.4;38.5] | [12.7;52.5] | [11.9;43.8] | [19.1;51.9] | [16.7;41.9] | [17.1;38.8] |
|  | vYF 4 Log | N | 18 | 18 | 13 | 13 | 16 | 17 | 18 |
|  |  | GM | 16.2 | 17.7 | 19.1 | 20.8 | 23.4 | 20.8 | 19.9 |
|  |  | 95% CI | [9.4;28.1] | [10.8;29.0] | [10.5;35.0] | [10.3;42.0] | [13.1;42.0] | [12.7;34.0] | [12.4;31.9] |
|  | vYF 5 Log | N | 18 | 18 | 13 | 12 | 18 | 17 | 18 |
|  |  | GM | 18.9 | 20.5 | 25.1 | 24.6 | 28.6 | 25.2 | 21.1 |
|  |  | 95% CI | [11.1;32.1] | [12.7;33.3] | [12.8;49.0] | [14.3;42.4] | [17.8;46.0] | [15.4;41.3] | [12.8;34.8] |
|  | vYF 6 Log | N | 18 | 17 | 11 | 11 | 18 | 16 | 18 |
|  |  | GM | 37.6 | 39.9 | 49.9 | 56.9 | 45.8 | 45.1 | 45.9 |
|  |  | 95% CI | [20.7;68.2] | [21.3;74.7] | [21.5;116.2] | [23.9;135.7] | [25.4;82.4] | [234.0;84.7] | [25.5;82.4] |
| Interleukin 18 (pg/mL) | YF-VAX | N | 18 | 18 | 13 | 12 | 18 | 18 | 18 |
|  |  | GM | 17 | 15 | 19 | 18 | 23 | 24 | 20 |
|  |  | 95% CI | [10.5;27.3] | [9.5;24.6] | [10.7;32.3] | [10.4;30.2] | [14.7;37.1] | [16.4;36.5] | [11.9;32.5] |
|  | vYF 4 Log | N | 18 | 18 | 13 | 13 | 16 | 17 | 18 |
|  |  | GM | 14.6 | 13.8 | 21.8 | 11.1 | 18.8 | 20.5 | 17.2 |
|  |  | 95% CI | [7.5;28.4] | [7.6;25.1] | [12.7;37.2] | [6.5;18.9] | [11.8;30.1] | [13.1;32.2] | [9.2;32.3] |
|  | vYF 5 Log | N | 18 | 18 | 13 | 12 | 18 | 17 | 18 |
|  |  | GM | 11.7 | 9.4 | 13.0 | 9.4 | 15.1 | 16.8 | 12.5 |
|  |  | 95% CI | [5.8;23.7] | [4.5;19.7] | [5.2;32.8] | [4.2;21.1] | [8.0;28.7] | [10.2;27.5] | [7.5;21.1] |
|  | vYF 6 Log | N | 18 | 17 | 11 | 11 | 18 | 16 | 18 |
|  |  | GM | 17.6 | 16.7 | 16.0 | 23.0 | 19.0 | 18.3 | 21.6 |
|  |  | 95% CI | [9.7;31.7] | [8.9;31.2] | [5.7;44.3] | [16.4;32.2] | [11.6;31.3] | [9.5;35.1] | [14.0;33.3] |
| Interleukin 5 (pg/mL) | YF-VAX | N | 18 | 18 | 13 | 12 | 18 | 18 | 18 |
|  |  | GM | 4 | 5 | 6 | 5 | 4 | 5 | 4 |
|  |  | 95% CI | [2.5;7.6] | [2.8;9.4] | [3.1;13.4] | [2.4;10.1] | [2.4;7.8] | [2.7;8.2] | [2.2;7.2] |
|  | vYF 4 Log | N | 18 | 18 | 13 | 13 | 16 | 17 | 18 |
|  |  | GM | 2.8 | 2.7 | 3.6 | 3.2 | 2.7 | 2.6 | 2.8 |
|  |  | 95% CI | [1.7;4.9] | [1.5;4.7] | [1.7;7.4] | [1.7;6.3] | [1.5;4.8] | [1.5;4.6] | [1.7;4.5] |
|  | vYF 5 Log | N | 17 | 18 | 13 | 12 | 18 | 17 | 18 |
|  |  | GM | 2.9 | 2.4 | 2.2 | 2.5 | 2.1 | 2.2 | 2.0 |
|  |  | 95% CI | [1.7;5.1] | [1.2;4.5] | [0.9;5.0] | [1.3;4.9] | [1.0;4.7] | [1.0;4.8] | [1.0;4.3] |
|  | vYF 6 Log | N | 18 | 17 | 11 | 11 | 18 | 16 | 18 |
|  |  | GM | 7.9 | 8.2 | 6.9 | 9.5 | 7.4 | 8.6 | 6.9 |
|  |  | 95% CI | [4.7;13.3] | [4.6;14.6] | [3.6;13.1] | [3.1;28.7] | [3.7;14.9] | [4.9;15.1] | [3.4;14.2] |
| Interleukin 6 (pg/mL) | YF-VAX | N | 18 | 18 | 13 | 12 | 18 | 18 | 18 |
|  |  | GM | 0 | 1 | 1 | 0 | 1 | 0 | 0 |
|  |  | 95% CI | [0.1;0.5] | [0.2;1.4] | [0.2;2.0] | [0.2;1.4] | [0.3;1.5] | [0.1;0.9] | [0.1;0.6] |
|  | vYF 4 Log | N | 18 | 18 | 13 | 13 | 16 | 17 | 18 |
|  |  | GM | 0.6 | 0.5 | 0.8 | 0.5 | 0.5 | 0.4 | 0.5 |
|  |  | 95% CI | [0.3;1.3] | [0.2;1.2] | [0.3;1.9] | [0.2;1.3] | [0.2;1.3] | [0.2;0.7] | [0.2;1.1] |
|  | vYF 5 Log | N | 17 | 18 | 13 | 12 | 18 | 17 | 18 |
|  |  | GM | 0.3 | 0.2 | 0.6 | 0.4 | 0.5 | 0.3 | 0.4 |
|  |  | 95% CI | [0.1;0.7] | [0.1;0.5] | [0.2;1.8] | [0.1;1.4] | [0.2;1.3] | [0.1;0.9] | [0.2;1.1] |
|  | vYF 6 Log | N | 18 | 17 | 11 | 11 | 18 | 16 | 18 |
|  |  | GM | 0.6 | 0.5 | 0.6 | 0.6 | 1.1 | 0.7 | 0.8 |
|  |  | 95% CI | [0.0;1.1] | [0.2;0.9] | [0.2;1.5] | [0.2;1.5] | [0.6;2.0] | [0.3;1.4] | [0.4;1.7] |
| Interleukin 8 (pg/mL) | YF-VAX | N | 18 | 18 | 13 | 12 | 18 | 18 | 18 |
|  |  | GM | 5 | 3 | 4 | 3 | 3 | 4 | 3 |
|  |  | 95% CI | [2.7;7.5] | [1.6;4.8] | [2.2;5.8] | [1.9;4.4] | [2.1;5.6] | [2.3;5.7] | [1.7;4.8] |
|  | vYF 4 Log | N | 18 | 18 | 13 | 13 | 16 | 17 | 18 |
|  |  | GM | 4.1 | 2.8 | 3.2 | 2.7 | 3.2 | 3.7 | 3.2 |
|  |  | 95% CI | [2. 8;6.2] | [2.1;3.8] | [2.5;4.2] | [2.0;3.7] | [2.6;3.9] | [2.7;5.0] | [2.2;4.6] |
|  | vYF 5 Log | N | 18 | 18 | 13 | 12 | 18 | 17 | 18 |
|  |  | GM | 4.2 | 2.7 | 3.3 | 2.7 | 3.1 | 3.7 | 2.8 |
|  |  | 95% CI | [2.8;6.4] | [2.2;3.3] | [2.2;4.8] | [1.9;3.9] | [2.4;4.1] | [3.0;4.5] | [1.9;4.2] |
|  | vYF 6 Log | N | 18 | 17 | 11 | 11 | 18 | 16 | 18 |
|  |  | GM | 6.0 | 3.7 | 4.6 | 5.6 | 4.7 | 4.5 | 5.8 |
|  |  | 95% CI | [4.1;8.9] | [2.2;6.0] | [2.4;9.0] | [3.2;9.8] | [2.9;7.6] | [2.9;6.9] | [4.1;8.1] |
| Interleukin-17A (pg/mL) | YF-VAX | N | 18 | 18 | 13 | 12 | 18 | 18 | 18 |
|  |  | GM | 1 | 1 | 1 | 1 | 1 | 1 | 1 |
|  |  | 95% CI | [0.5;1.7] | [0.5;1.8] | [0.6;3.6] | [0.5;2.0] | [0.6;2.1] | [0.5;1.8] | [0.5;1.8] |
|  | vYF 4 Log | N | 18 | 18 | 13 | 13 | 16 | 17 | 18 |
|  |  | GM | 1.3 | 1.3 | 1.4 | 1.6 | 1.4 | 1.3 | 1.3 |
|  |  | 95% CI | [0.7;2.6] | [0.7;2.6] | [0.6;3.1] | [0.7;3.9] | [0.7;2.9] | [0.7;2.4] | [0.7;2.3] |
|  | vYF 5 Log | N | 18 | 18 | 13 | 12 | 18 | 17 | 18 |
|  |  | GM | 1.4 | 1.0 | 1.1 | 1.3 | 1.2 | 1.1 | 1.3 |
|  |  | 95% CI | [0.7;2.6] | [0.6;1.8] | [0.6;1.8] | [0.5;3.3] | [0.7;2.3] | [0.6;2.2] | [0.7;2.6] |
|  | vYF 6 Log | N | 18 | 17 | 11 | 11 | 18 | 16 | 18 |
|  |  | GM | 2 | 1.9 | 1.8 | 3.8 | 2.5 | 1.7 | 2.6 |
|  |  | 95% CI | [1.0;4.0] | [0.9;3.9] | [0.7;4.8] | [1.5;9.7] | [1.2;5.1] | [0.8;3.6] | [1.2;5.6] |
| Macrophage Inflammatory Protein 1 Alpha (pg/mL) | YF-VAX | N | 18 | 18 | 13 | 12 | 18 | 18 | 18 |
|  |  | GM | 6 | 5 | 7 | 7 | 5 | 5 | 5 |
|  |  | 95% CI | [2.9;11.0] | [2.5;9.3] | [2.8;17.7] | [2.8;15.1] | [2.6;10.0] | [2.6;9.0] | [2.6;9.4] |
|  | vYF 4 Log | N | 18 | 18 | 13 | 13 | 16 | 17 | 18 |
|  |  | GM | 4.5 | 4.3 | 4.1 | 5.0 | 4.4 | 4.1 | 3.9 |
|  |  | 95% CI | [2.5;8.3] | [2.3;7.9] | [2.1;8.2] | [2.2;11.1] | [2.3;8.3] | [2.4;7.0] | [2.3;6.6] |
|  | vYF 5 Log | N | 18 | 18 | 13 | 12 | 18 | 17 | 18 |
|  |  | GM | 4.8 | 4.2 | 4.1 | 3.8 | 4.8 | 3.8 | 3.8 |
|  |  | 95% CI | [2.6;9.0] | [2.3;7.5] | [2.1;8.1] | [1.8;7.9] | [2.6;9.1] | [2.1;6.9] | [2.1;6.7] |
|  | vYF 6 Log | N | 18 | 17 | 11 | 11 | 18 | 16 | 18 |
|  |  | GM | 10.6 | 10.2 | 10.0 | 23.4 | 11.5 | 11.6 | 10.7 |
|  |  | 95% CI | [5.2;21.6] | [4.8;21.7] | [3.8;26.3] | [10.5;52.4] | [5.5;24.3] | [5.2;25.8] | [4.8;23.9] |
| Monocyte Chemotactic Protein 1 (pg/mL) | YF-VAX | N | 18 | 18 | 13 | 12 | 18 | 18 | 18 |
|  |  | GM | 511 | 543 | 426 | 626 | 585 | 516 | 454 |
|  |  | 95% CI | [398.2;656.4] | [425.6;693.3] | [336.8;538.0] | [460.2;850.7] | [485.4;705.2] | [417.2;637.9] | [368.5;559.2] |
|  | vYF 4 Log | N | 18 | 18 | 13 | 13 | 16 | 17 | 18 |
|  |  | GM | 390.8 | 412.7 | 404.6 | 456.8 | 493.8 | 429.0 | 396.1 |
|  |  | 95% CI | [304.4;501.8] | [317.2;537.0] | [291.1;562.3] | [338.9;615.6] | [362.1;673.3] | [326.9;562.9] | [296.1;529.9] |
|  | vYF 5 Log | N | 18 | 18 | 13 | 12 | 18 | 17 | 18 |
|  |  | GM | 432.1 | 444.5 | 407.4 | 445.3 | 504.4 | 453.1 | 395.6 |
|  |  | 95% CI | [328.6;568.1] | [349.3;565.5] | [300.4;552.6] | [325.8;608.6] | [397.2;640.7] | [354.4;579.4] | [319.1;490.3] |
|  | vYF 6 Log | N | 18 | 17 | 11 | 11 | 18 | 16 | 18 |
|  |  | GM | 522.1 | 579.5 | 519.2 | 582.7 | 722.8 | 544.1 | 505.6 |
|  |  | 95% CI | [440.2;619.2] | [484.8;692.8] | [428.4;629.4] | [443.9;764.9] | [596.0;876.5] | [463.4;638.9] | [425.4;600.9] |
| Tumor Necrosis Factor (pg/mL) | YF-VAX | N | 18 | 18 | 13 | 12 | 18 | 18 | 18 |
|  |  | GM | 19 | 19 | 22 | 19 | 23 | 19 | 18 |
|  |  | 95% CI | [12.0;29.2] | [12.0;29.0] | [12.6;36.7] | [10.1;37.5] | [13.5;38.6] | [12.0;29.3] | [11.3;28.1] |
|  | vYF 4 Log | N | 18 | 18 | 13 | 13 | 16 | 17 | 18 |
|  |  | GM | 14.8 | 15.2 | 16.6 | 14.7 | 18.6 | 16.0 | 14.9 |
|  |  | 95% CI | [11.0;19.8] | [11.1;20.9] | [13.0;21.1] | [9.5;22.6] | [13.8;25.2] | [13.2;19.4] | [12.2;18.4] |
|  | vYF 5 Log | N | 18 | 18 | 13 | 12 | 18 | 17 | 18 |
|  |  | GM | 11.9 | 11.6 | 10.5 | 16.9 | 14.4 | 13.8 | 11.9 |
|  |  | 95% CI | [7.6;18.6] | [7.6;17.7] | [7.1;15.7] | [9.6;29.7] | [8.9;23.2] | [8.5;22.6] | [6.8;20.2] |
|  | vYF 6 Log | N | 18 | 17 | 11 | 11 | 18 | 16 | 18 |
|  |  | GM | 20.6 | 19.6 | 21.9 | 25.8 | 26.5 | 19.4 | 22.5 |
|  |  | 95% CI | [14.5;29.3] | [13.2;29.0] | [12.1;39.6] | [13.7;48.4] | [18.1;38.7] | [11.4;33.1] | [13.8;36.7] |

Figure S1. Individual participant time course for IFI27 gene expression by vaccine and dose group (DDCt presented correspond to a log_2_ fold change in the situation of 100% qPCR reaction efficiency) (FAS).

**
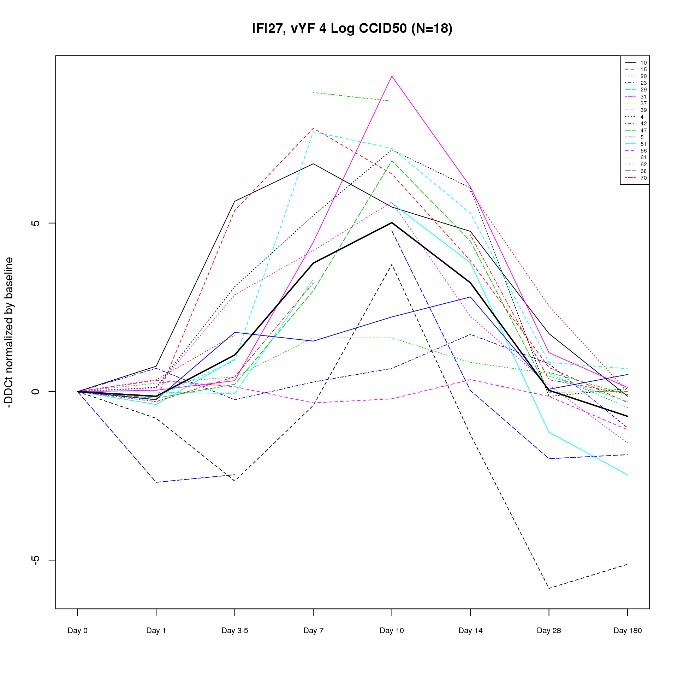

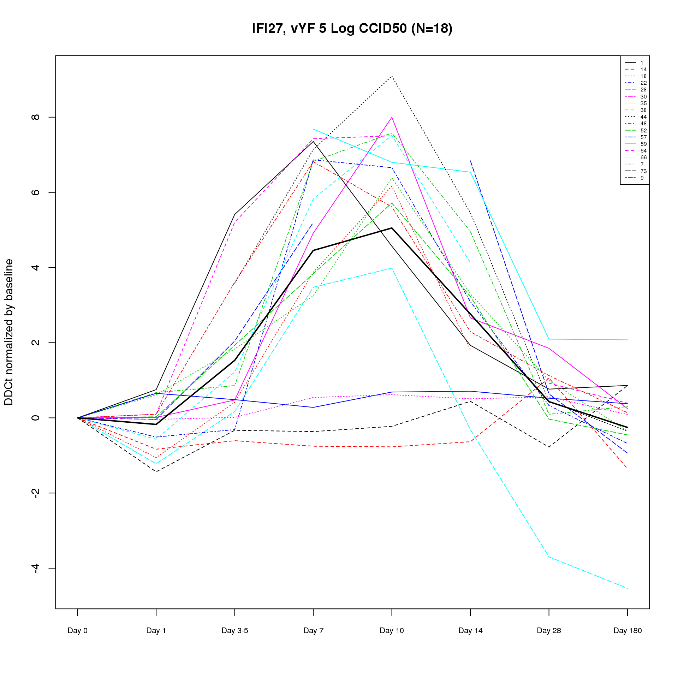
**

**
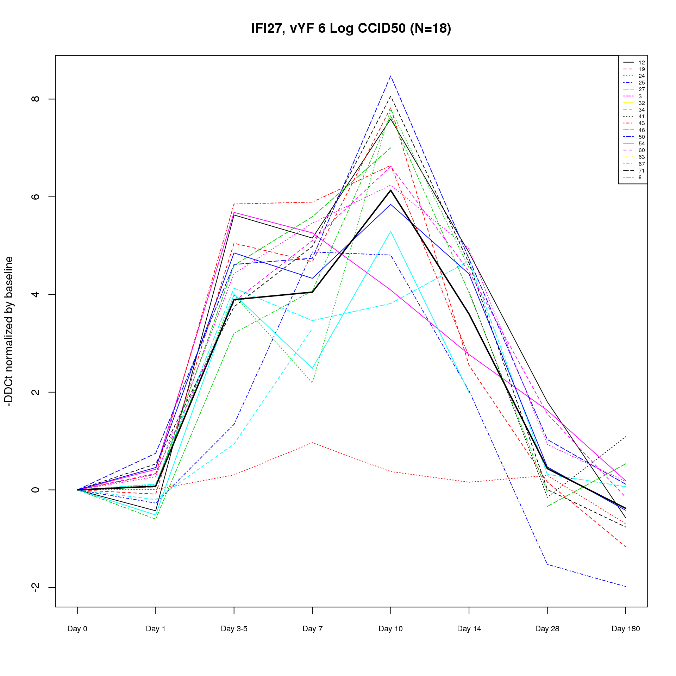

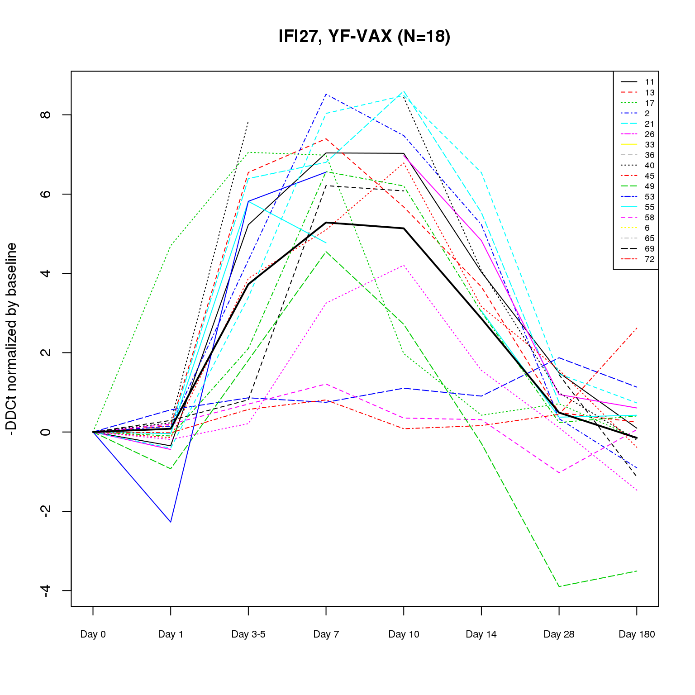
**

# **References**

1. Jury EC, Kabouridis PS. New role for Agrin in T cells and its potential importance in immune system regulation. *Arthritis Res Ther* 2010; **12**(2): 205.

2. Balas C, Kennel A, Deauvieau F, et al. Different innate signatures induced in human monocyte-derived dendritic cells by wild-type dengue 3 virus, attenuated but reactogenic dengue 3 vaccine virus, or attenuated nonreactogenic dengue 1-4 vaccine virus strains. *J Infect Dis* 2011; **203**(1): 103–8.

3. Gizzi AS, Grove TL, Arnold JJ, et al. A naturally occurring antiviral ribonucleotide encoded by the human genome. *Nature* 2018; **558**(7711): 610–4.

4. Fusco DN, Pratt H, Kandilas S, et al. HELZ2 Is an IFN Effector Mediating Suppression of Dengue Virus. *Front Microbiol* 2017; **8**: 240.

5. Perng YC, Lenschow DJ. ISG15 in antiviral immunity and beyond. *Nat Rev Microbiol* 2018; **16**(7): 423–39.

6. Liu Z, Xiang Y, Sun G. The KCTD family of proteins: structure, function, disease relevance. *Cell Biosci* 2013; **3**(1): 45.

7. Papic N, Maxwell CI, Delker DA, Liu S, Heale BS, Hagedorn CH. RNA-sequencing analysis of 5' capped RNAs identifies many new differentially expressed genes in acute hepatitis C virus infection. *Viruses* 2012; **4**(4): 581–612.

8. Yu J, Liu SL. Emerging Role of LY6E in Virus-Host Interactions. *Viruses* 2019; **11**(11).

9. Atasheva S, Frolova EI, Frolov I. Interferon-stimulated poly(ADP-Ribose) polymerases are potent inhibitors of cellular translation and virus replication. *J Virol* 2014; **88**(4): 2116–30.

10. Li L, Zhao H, Liu P, et al. PARP12 suppresses Zika virus infection through PARP-dependent degradation of NS1 and NS3 viral proteins. *Sci Signal* 2018; **11**(535).

11. Duschene KS, Broderick JB. Viperin: a radical response to viral infection. *Biomol Concepts* 2012; **3**(3): 255–66.

12. Zheng Q, Hou J, Zhou Y, Yang Y, Xie B, Cao X. Siglec1 suppresses antiviral innate immune response by inducing TBK1 degradation via the ubiquitin ligase TRIM27. *Cell Res* 2015; **25**(10): 1121–36.

13. Gupta N, Rao PV. Transcriptomic profile of host response in Japanese encephalitis virus infection. *Virol J* 2011; **8**: 92.

14. Prestwood TR, May MM, Plummer EM, Morar MM, Yauch LE, Shresta S. Trafficking and replication patterns reveal splenic macrophages as major targets of dengue virus in mice. *J Virol* 2012; **86**(22): 12138–47.

15. Liu F, Zhou P, Wang Q, Zhang M, Li D. The Schlafen family: complex roles in different cell types and virus replication. *Cell Biol Int* 2018; **42**(1): 2–8.

16. Mavrommatis E, Fish EN, Platanias LC. The schlafen family of proteins and their regulation by interferons. *J Interferon Cytokine Res* 2013; **33**(4): 206–10.

17. Nakaya HI, Li S, Pulendran B. Systems vaccinology: learning to compute the behavior of vaccine induced immunity. *Wiley Interdiscip Rev Syst Biol Med* 2012; **4**(2): 193–205.

18. Francois-Newton V, Magno de Freitas Almeida G, Payelle-Brogard B, et al. USP18-based negative feedback control is induced by type I and type III interferons and specifically inactivates interferon alpha response. *PLoS One* 2011; **6**(7): e22200.

19. Mladinich MC, Schwedes J, Mackow ER. Zika Virus Persistently Infects and Is Basolaterally Released from Primary Human Brain Microvascular Endothelial Cells. *mBio* 2017; **8**(4).

20. Chiu HP, Chiu H, Yang CF, et al. Inhibition of Japanese encephalitis virus infection by the host zinc-finger antiviral protein. *PLoS Pathog* 2018; **14**(7): e1007166.
